# Reversion mutations in phosphoprotein P of a codon-pair-deoptimized human respiratory syncytial virus confer increased transcription, immunogenicity, and genetic stability without loss of attenuation

**DOI:** 10.1371/journal.ppat.1010191

**Published:** 2021-12-29

**Authors:** Jessica W. Chen, Lijuan Yang, Celia Santos, Sergio A. Hassan, Peter L. Collins, Ursula J. Buchholz, Cyril Le Nouën

**Affiliations:** 1 RNA Viruses Section, Laboratory of Infectious Diseases, NIAID, NIH, Bethesda, Maryland, United States of America; 2 Bioinformatics and Computational Biosciences Branch, NIAID, NIH, Bethesda, Maryland, United States of America; Virginia Tech: Virginia Polytechnic Institute and State University, UNITED STATES

## Abstract

Recoding viral genomes by introducing numerous synonymous nucleotide substitutions that create suboptimal codon pairs provides new live-attenuated vaccine candidates. Because recoding typically involves a large number of nucleotide substitutions, the risk of de-attenuation is presumed to be low. However, this has not been thoroughly studied. We previously generated human respiratory syncytial virus (RSV) in which the NS1, NS2, N, P, M and SH ORFs were codon-pair deoptimized (CPD) by 695 synonymous nucleotide changes (Min A virus). Min A exhibited a global reduction in transcription and protein synthesis, was restricted for replication *in vitro* and *in vivo*, and exhibited moderate temperature sensitivity. Here, we show that under selective pressure by serial passage at progressively increasing temperatures, Min A regained replication fitness and lost its temperature sensitivity. Whole-genome deep sequencing identified numerous missense mutations in several genes, in particular ones accumulating between codons 25 and 34 of the phosphoprotein (P), a polymerase cofactor and chaperone. When re-introduced into Min A, these P mutations restored viral transcription to wt level, resulting in increased protein expression and RNA replication. Molecular dynamic simulations suggested that these P mutations increased the flexibility of the N-terminal domain of P, which might facilitate its interaction with the nucleoprotein N, and increase the functional efficiency of the RSV transcription/replication complex. Finally, we evaluated the effect of the P mutations on Min A replication and immunogenicity in hamsters. Mutation P[F28V] paradoxically reduced Min A replication but not its immunogenicity. The further addition of one missense mutation each in M and L generated a version of Min A with increased genetic stability. Thus, this study provides further insight into the adaptability of large-scale recoded RNA viruses under selective pressure and identified an improved CPD RSV vaccine candidate.

## Author summary

Synonymous recoding of viral genomes by codon-pair deoptimization (CPD) generates live-attenuated vaccines presumed to be genetically stable due to the high number of nucleotide substitutions. However, their actual genetic stability under selective pressure was largely unknown. In a recoded human respiratory syncytial virus (RSV) mutant called Min A, six of 11 ORFs were CPD, reducing protein expression and inducing moderate temperature sensitivity and attenuation. When passaged *in vitro* under selective pressure, Min A lost its temperature-sensitive phenotype and regained fitness by the acquisition of numerous mutations, in particular missense mutations in the viral phosphoprotein (P), a polymerase cofactor and a chaperone for soluble nucleoprotein. These P mutations increased RSV gene transcription globally, thereby increasing RSV protein expression, RNA replication, and virus particle production. Thus, the P mutations increased the efficiency of the RSV transcription/replication complex, compensating for the reduced protein expression due to CPD. In addition, introduction of the P mutations into Min A generated a live-attenuated vaccine candidate with increased genetic stability. Surprisingly, this vaccine candidate exhibited increased attenuation and, paradoxically, exhibited increased immunogenicity per plaque-forming unit in hamsters. This study provides insights into the adaptability of large-scale recoded RNA viruses and identified an improved CPD RSV vaccine candidate.

## Introduction

Genome recoding by the introduction of a large number of synonymous codon changes designed to deoptimize viral coding sequences is gaining increasing use as a strategy to attenuate pathogens and create candidate live vaccines. In particular, recoding of ORFs by codon-pair deoptimization (CPD) has emerged as a highly efficient attenuation strategy.

Most amino acids are encoded by more than one codon, and the various codons encoding any particular amino acid typically appear with frequencies that differ from a random distribution in a species-specific fashion. Similarly, codon-pair combinations in open reading frames (ORFs) occur more or less frequently than would be expected in a random distribution [1,2]. CPD involves recoding ORFs to increase the content of codon pairs that are under-represented in the human genome and therefore are considered suboptimal. This typically is done without changing overall codon usage or the encoded amino acid sequence. The underlying mechanism of attenuation by CPD is not completely understood and might be multifactorial. For example, CPD has the potential to reduce translation efficiency by affecting mRNA secondary structure [3] or stability [4], and/or translation elongation [4,5]. Another hypothesis proposes that attenuation relies on the immunomodulatory effects of the increases in CpG or UpA content resulting from CPD [6].

Attenuation of pathogens by CPD offers several advantages for vaccine development. Because amino acid sequences remain unchanged, the resulting vaccine candidates should express the full array of antigenic epitopes. Interestingly, previous studies have shown that the immunogenicity of recoded viruses was frequently unchanged compared to the parental wt virus despite reduced virus replication *in vivo* [3,6,7]. The level of pathogen attenuation can theoretically be adjusted by increasing or decreasing the extent of recoding. Relevant to RNA viruses with high mutations rates, large-scale recoding should reduce the rate and magnitude of reversion or de-attenuation due to the large number of nucleotide substitutions introduced, and are thought to result in extremely stable vaccine candidates [2,7–10]. However, this generally had not been rigorously tested.

Human respiratory syncytial virus (RSV) is a major worldwide respiratory pathogen responsible for severe lower respiratory infections in infants and young children, and in elderly or immunocompromised individuals [11–13]. RSV belongs to the *Pneumoviridae* family of the *Mononegavirales* order. It has a 15.2 kb, negative-sense, single-stranded RNA genome that contains 10 genes encoding the following 11 different proteins: nonstructural proteins 1 and 2 (NS1 and NS2), nucleoprotein N, phosphoprotein P; matrix M protein; small hydrophobic SH protein, attachment G glycoprotein, fusion F protein, RNA synthesis factors M2-1 and M2-2 encoded from overlapping ORFs in the M2 gene, and large polymerase L protein. The order of the 10 genes in the RSV genome is: 3′-NS1-NS2-N-P-M-SH-G-F-M2-L-5′, and the genome contains short extragenic leader and trailer regions at the 3’ and 5’ ends, respectively. The genes are transcribed sequentially from a single 3´-promoter into separate mRNAs, guided by short conserved gene-start and gene-end signals that flank each gene.

There is an urgent need for RSV vaccines suitable for use in young children and the elderly, but approved RSV vaccines are not yet available. Live-attenuated RSV vaccines are essential for pediatric use because they are not associated with priming for the disease enhancement in RSV-naïve recipients that has been observed in an early clinical study with a formalin-inactivated RSV vaccine [14–18] and with RSV subunit vaccines in animal models [19].

We previously created four RSVs containing different numbers of CPD ORFs. In one virus (Min FLC), nine of the 11 RSV ORFs were subjected to CPD (the M2-1 and M2-2 ORFs were left unchanged because of their complex mode of expression) [20,21]. A second virus (Min L) had CPD L [21]. A third (Min B) had CPD G and F [22]. The fourth (Min A) had CPD NS1, NS2, N, P, M, and SH. These CPD RSVs exhibited a range of attenuation *in vitro* and *in vivo*. Surprisingly, they were temperature sensitive. This temperature sensitivity provided a means to apply selective pressure to the CPD RSVs by a temperature stress test, in which the virus of interest is subjected to serial passage *in vitro* at incrementally-increasing temperatures. This creates conditions that favor the outgrowth and detection of viruses bearing mutations that reduce temperature sensitivity and likely are de-attenuating, providing a sensitive assay of genetic stability. Since a temperature gradient occurs along the human respiratory tract, and since respiratory virus infections progress from the upper to the lower respiratory tract, temperature stress is a relevant model to assess the genetic stability of live-attenuated respiratory virus vaccine candidates.

**Fig 1:**
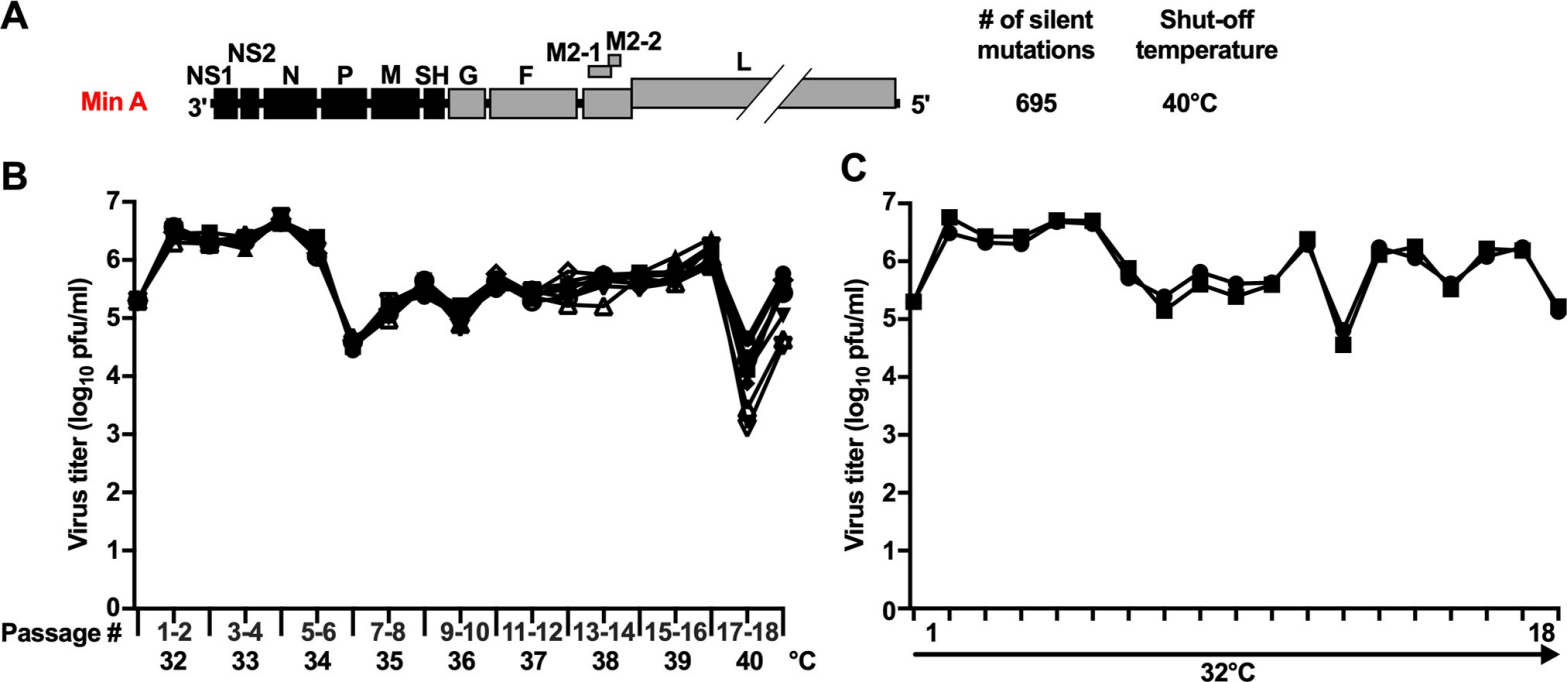
Min A was phenotypically unstable when serially passaged at increasing temperature. (A) Genome organization of Min A. CPD ORFs (NS1, NS2, N, P, M and SH) and wt ORFs (G, F, M2-1, M2-2 and L) are shown in black and grey, respectively. The number of silent nucleotide substitutions introduced during CPD and the shut-off temperature in Vero cells (T_SH_) are indicated. The T_SH_ is the lowest restrictive temperature at which there is a reduction in virus titer compared with the permissive temperature of 32°C that is 100-fold or greater than that of wt RSV at this temperature versus 32°C. (B-C) Temperature stress test. Eleven replicate cultures of Vero cells in 25 cm^2^ flasks were inoculated with an MOI of 0.1 PFU/cell of Min A and subjected to serial passage in parallel. Each flask represented a separate lineage. In nine lineages (B), the temperature of incubation was increased by one °C every second passage from 32°C to 40°C for a total of 18 passages. The other two lineages (C) were passaged 18 times at the permissive temperature of 32°C as controls. When cells had formed extensive syncytia or were starting to detach (typically between days six and 11), they were scraped into the medium and vortexed for 30s to detach virus, and the supernatants were clarified by low-speed centrifugation. Twenty per cent of the clarified supernatants (one ml of the five ml total) were used to inoculate the following passage. The remainder of the supernatants were aliquoted and snap frozen in dry ice for subsequent titration at 32°C and sequence analysis. The x-axis shows the passage number and associated temperatures, and the y-axis shows the virus titer of the initial inoculum and passage harvests. Each symbol represents one lineage.

In previous *in vitro* stress tests, Min FLC, which has a shut-off temperature (T_SH_, defined in the legend of Fig 1A) of 35°C, was found to be genetically and phenotypically stable, whereas Min L (T_SH_: 37°C) and Min B (T_SH_: 38°C) lost part of their ts phenotypes under selective pressure [20–22]. Min L and Min B each acquired a broad, variable array of mutations in CPD and, somewhat surprisingly, non-CPD genes. For example, in the case of Min L, mutations in the RNA synthesis factor M2-1, which had not been CPD, were found to substantially restore defective transcription of the CPD L gene. Introduction of these mutations into Min L substantially restored virus fitness *in vitro* and, in case of one of these two mutations, also *in vivo*. These findings were used to modify Min L, yielding a virus that had increased genetic stability and paradoxically had both increased attenuation and increased immunogenicity, thus providing an improved vaccine candidate that is presently being evaluated in Phase 1 clinical studies (ClinicalTrials.gov number NCT04295070) [21]. However, the development of an RSV vaccine using the CPD strategy ideally should not depend on a single vaccine candidate, as past experience has taught us that the identification of suitable levels of attenuation and immunogenicity for a live-attenuated RSV vaccine ideally depends on evaluation of more than one candidate.

In the present study, we assayed the genetic stability of Min A, bearing CPD NS1, NS2, N, P, M and SH ORFs, by applying a temperature stress test. Similar to the findings with Min L, we found that during serial passage at increasing temperatures, Min A acquired a broad, variable array of nucleotide substitutions, predominantly missense mutations, in multiple genes, and lost much of its temperature sensitivity. In case of Min A, prominent mutations targeted the phosphoprotein P, which is a polymerase cofactor that tethers L to the nucleocapsid [23–25]. P is also a chaperonin that maintains soluble nucleoprotein in a monomeric RNA-free state [26–28], and recruits the M2-1 processivity factor to the polymerase complex [29–31], among other activities.

Molecular dynamic simulations suggested that these P mutations increased the flexibility of P, which was predicted to modify its interaction with N. In addition, when reintroduced into Min A, some of these mutations increased attenuation without reducing the immunogenicity of Min A in the hamster model, which ultimately allowed us to identify a Min A-derived virus that was more attenuated than Min A, but exhibited increased immunogenicity per PFU and increased genetic stability, thus providing an improved vaccine candidate for RSV.

## Results

### Min A was phenotypically unstable during passage at increasing temperatures

Min A is a previously-described derivative of RSV strain A2 [32] in which the first six ORFs in the genome, namely NS1, NS2, N, P, M, and SH, were subjected to CPD; this introduced, respectively, 65, 60, 241, 143, 163 and 23 synonymous nucleotide substitutions, for a total of 695 (Fig 1A) [20]. Amino acid sequences and codon usage were unchanged. Min A is moderately temperature-sensitive with a temperature shut-off (T_SH_; defined in the legend to Fig 1A) for efficient viral replication in cell culture of 40°C [20], while wt RSV replicates efficiently at 40°C.

We subjected Min A to an *in vitro* temperature stress test. Eleven 25 cm^2^ flasks of Vero cells were inoculated with Min A at an initial multiplicity of infection (MOI) of 0.1 plaque forming unit (PFU) per cell. Each flask represented a separate passage lineage. Nine lineages were subjected to 18 passages in parallel beginning at 32°C and proceeding by one °C increases to 40°C, with two passages at each temperature, representing four months of continuous culture. The remaining two lineages were passaged in parallel 18 times at the permissive temperature of 32°C as controls. After each passage, clarified culture supernatants were aliquoted and snap frozen in dry ice for subsequent titration and sequencing by Sanger sequencing and/or deep sequencing.

For each of the nine Min A lineages incubated at increasing temperature, viral titers of P1 to P5 (the first passage at 34°C) ranged between 10^6^ and 10^6.5^ PFU/ml (Fig 1B). From P5 to P6 (34°C), titers in each lineage decreased about 100-fold to 10^4.5^ PFU/ml. Then, from P6 to P16 (second passage at 39°C), titers in each lineage gradually increased to reach approximately 10^6^ PFU/ml. During the first passage at 40°C (P17), titers sharply decreased about 20- to 1000-fold in every lineage, ranging from 10^3^ to 10^4.7^ PFU/ml. Finally, during the second passage at 40°C (P18), titers in each lineage increased to reach between 10^4.5^ and 10^5.8^ PFU/ml, showing that each Min A lineage had lost some temperature sensitivity and restriction. Min A titers in the control flasks at 32°C fluctuated between 10^6.8^ and 10^4.6^ PFU/ml and reached approximately 10^5^ PFU/ml at the last passage (P18, Fig 1C).

### Multiple prominent mutations were selected during passage at increasing temperature

To evaluate genetic stability under temperature stress, whole-genome deep sequencing was performed on viral RNA for each of the nine lineages passaged at increasing temperature (lineages #1–9) and the two controls (Ct1, Ct2) from the last passage (P18) of the stress test shown in Fig 1. Whole genome sequencing revealed that each of lineages #1–9 had accumulated between three and seven prominent mutations (defined as being present in ≥45% of the reads) during passage, while each of the two control lineages passaged at 32°C contained a single prominent change (Table 1). In total, the 11 lineages had 43 prominent mutations, of which 36 were different (Table 1).

**Table 1:** Mutations detected at a frequency of ≥45% in each of the nine Min A lineages at the end of the temperature stress test (passage 18) as well as in each of the two controls.

			Lineage no.*										
Gene	nt mutation	aa mutation	1	2	3	4	5	6	7	8	9	Ct1	Ct2
NS2 5’UTR	t612c	/					91						
N 5’UTR	a1138g	/				59							
N	c1301a^†^	T54N								49			
N	a1459c^†^	K107Q		57									
N	a1749g^†^^‡^^§^	K203K (silent)				94							
N	t2151c^†^^‡^^§^	Y337Y (silent)								62			
P gene start	c2334t	/					68						
P	t2364c	P6P (silent)							64		61		
P	a2376t	G10G (silent)				77							
P	t2388c^†^^‡^^§^	N14N (silent)									45		
P	a2420c	K25T	98	94									
P	g2421t	K25N										58	49
P	g2427t^†^^‡^	K27N				97				62			
P	t2428g^†^	F28V					98				53		
P	t2428c^†^, t2429c^†^	F28P							58				
P	a2441c	K32T			93								
P	c2446t^†^	P34S			92								
P	t2577c^†^^‡^^§^	P77P (silent)							73				
P	g3032a^†^	G229E			91								
P	a3046c^†^	N234H	78										
P 3’UTR	a3195g	/					89						
M	a3629t^†^	K123M					62						
SH-G intergenic	a4662g	/								53			
M2-1	a7754g	M50V									78		
M2-1	a8091g	K162R						54					
L	t8950c, t8951c	V151A				59							
L	t9453c	C319R						56					
L	t10622c	F708F (silent)								45			
L	a10782g	N762D	77										
L	a11361t	I955L								49			
L	g11574a	D1026N	99	97									
L	g13194a	V1566I							69				
L	a13208g	I1570M								58			
L	a13898g	I1800M							69				
L	t14669c	F2057F (silent)						68					
L	t14748c	S2084P			62		93						

“/” indicates that the amino acid mutation is not applicable for this particular mutation as the given mutation is localized in a non-translated region.

*Percentage of reads with the indicated mutation; only mutations present in ≥45% of the reads are shown.

Mutations identified between aa 25 and 34 inclusive of P are highlighted by a grey shading.

Note that mutations t2428c and t2429c, and mutations t8950c and t8951c were each colocalized on the same viral genomes.

Nucleotide numbering is based on RSV sequence KT992094.

^†^Mutations involving a codon that had been changed as part of CPD of NS1, NS2, N, P, M, or SH.

^‡^Mutations involving a nucleotide that had been changed as part of CPD of NS1, NS2, N, P, M, or SH.

^§^Mutation involving a nucleotide that had been changed as part of CPD of NS1, NS2, N, P, M, or SH and that restored wt sequence.

Most of the 36 different prominent mutations did not represent reversions from CPD nucleotide assignments back to wt assignments. Only 18 occurred in ORFs that had been subjected to CPD, specifically in N, P, and M. Of these, only five involved nucleotide positions whose assignments had been changed as part of CPD; of these, four reverted to the original nucleotide assignments while the fifth changed to a different nucleotide and created a missense mutation (g2427t, K27N in P).

Of the 36 different prominent mutations, 31 (86%) were within ORFs and only five (14%) were not. Of these five mutations outside of ORFs, one (a4662g, lineage #8) occurred in an intergenic region (SH-G) and was considered insignificant because of the variable nature of the RSV intergenic regions. Another one was a c2334t substitution in the P gene start signal (GGGGCAAAT, lineage #5), which previously had been shown to have no detectable effect on transcription in a mini-genome system [33]. The other four mutations occurring outside of ORFs (lineages #4 and #5) were evaluated in a later section and found to be insignificant.

Out of the 31 different mutations occurring within ORFs, 23 (76%) were missense mutations, suggesting an overall bias toward amino acid change. The eight synonymous mutations within ORFs (labeled “silent” in Table 1) were considered insignificant because the ORFs involved are thought to lack RNA signals. Of the 23 different missense mutations, 17 each occurred in single lineages, while six each occurred in two lineages. The 23 different prominent missense mutations occurred in five ORFs: N, P, M, M2-1 and L, three of which were CPD (N, P and M). Most (78%) of the different prominent missense mutations were in P and L, with nine distinct mutations each. In this report, we focused on the P mutations.

Interestingly, seven of the nine unique missense mutations in P were localized in its N-terminal region and involved only five different aas (25, 27, 28, 32 and 34, see grey shading in Table 1). Nine of the 11 lineages had a single prominent P missense mutation at one of these five aa positions, while one lineage (#3) contained two. In addition, as indicated above, some of the P mutations occurred in more than a single lineage. Specifically, the mutation [K25T] was found in lineage #1 and #2, the mutation [K27N] in lineage #4 and #8, and the mutation [F28V] in lineage #5 and #9. The mutation [K25N] was common to both control lineages Ct1 and Ct2, showing that even without temperature stress, this region of the P ORF of Min A was prone to coding changes. Furthermore, P amino acid 25 was involved in two different prominent mutations, [K25T] and [K25N]; amino acid 27 in two different prominent mutations, [K27E] and [K27N]; and amino acid 28 in three different prominent mutations, [F28V], [F28I], and [F28P]. No prominent mutations in P were found in lineage #6. However, using a lower window (mutations identified as ≥5% to <45% of the reads, S1 Table), we found that lineage #6 contained a unique subdominant [F28I] mutation identified in 24% of the reads (S1 Table).

Using the lower window of ≥5% to <45% of reads (S1 Table), the 11 lineages were found to contain 58 additional mutations (including P[F28I] noted in the preceding sentence). These were designated subdominant mutations. They were distributed among all of the ORFs. Fifty-five of these subdominant mutations were different; only three were found in more than a single lineage (in each case, two lineages). Thirty (57%) were missense mutations, confirming the overall bias toward amino acid change. These 30 different subdominant missense mutations included four in the region of amino acids 25 to 34 in the P protein highlighted in Tables 1 and S1: [K25E] in Ct2, [K27E] in lineage #8 and Ct1, [F28V] in lineage #8, and [F28I] in lineage #6. Taken together, the whole-genome deep sequencing data suggested that the selective pressure during the stress test specifically favored the replication of virus bearing specific mutations in the N-terminal region of the P protein, particularly between aa 25 and 34.

### The P mutations identified between aa 25 and 34 were associated with a loss of temperature sensitivity by Min A

We next investigated potential contributions by the P protein missense mutations in the loss of the ts phenotype of Min A during the stress test experiment in Fig 1. We chose six prominent P missense mutations that each was present in two lineages at a level of ≥45% of reads, and/or were present in a single lineage at a level of ≥90% of reads (Tables 1 and S1). The six P missense mutations were: [K25T], [K25N], [K27N], [F28V], [K32T] and [P34S]. These were re-introduced individually by site-directed mutagenesis into the Min A antigenomic cDNA and rescued by reverse genetics, and the complete genome sequences were confirmed by Sanger sequencing.

The temperature sensitivity of the Min A-derived viruses was compared to Min A and wt RSV (Table 2). In this study, the titer of Min A at 40°C was 2.3 log_10_ lower than at 32°C, whereas the titer of wt RSV at 40°C was 0.4 log_10_ lower than at 32°C. Thus, the difference in the reduction in titer of Min A compared to wt RSV at the same temperatures was 1.9 log_10_, which was slightly less than the difference of ≥2.0 log_10_ that formally defines the temperature-sensitive phenotype (see legend of Fig 1). A 2.6 log_10_ difference was previously observed [20]. Although the temperature difference in the present study did not reach the threshold of temperature sensitivity, it was sufficient to evaluate possible effects of the mutations. We found that most of the P missense mutations that we had introduced into Min A substantially increased its ability to form plaques at 40°C. In particular, Min A containing the mutation P[K25T], P[K27N], or P[K32T] had titers at 40°C that were only 0.3–0.4 log_10_ lower than at 32°C, similar to wt RSV. In contrast, mutation P[P34S] was the least effective in compensating for the temperature sensitivity of Min A, resulting in a titer that was 1.7 log_10_ lower at 40°C than at 32°C.

**Table 2:** Temperature sensitivity of the CPD RSVs on Vero cells.

Virus titer (log_10_ PFU per ml) at indicated temperature (°C)								
Virus	32	35	36	37	38	39	40	T_SH_
Min A	6.2	6.5	6.4	6.1	6.1	5.1	3.9	**>40**
Min A-P[K25T]	7.0	7.0	7.0	7.0	6.9	6.8	6.6	**>40**
Min A-P[K25N]	7.0	7.0	6.9	6.9	6.8	6.7	5.5	**>40**
Min A-P[K27N]	6.8	6.8	6.8	6.8	6.8	6.6	6.5	**>40**
Min A-P[F28V]	6.7	6.8	6.8	6.7	6.8	6.6	6.0	**>40**
Min A-P[K32T]	6.9	7.0	6.9	6.9	6.8	6.9	6.8	**>40**
Min A-P[P34S]	5.7	5.7	5.5	5.2	6.0	4.7	4.0	**>40**
wt RSV	6.9	6.9	6.9	6.9	6.8	6.8	6.5	**>40**

The temperature sensitivity (ts) phenotype of the indicated viruses was evaluated by their efficiency to form plaques at 32°C, 35°C, 36°C, 37°C, 38°C, 39°C, and 40°C as previously described (21). Briefly, virus stocks were 10-fold serially diluted in OptiMEM and inoculated in duplicate in 24-well plates of Vero cells for two h at 32°C. A 0.8% methyl cellulose overlay in Leibovitz (L-15) media was added and plates were incubated for seven days in sealed caskets at the temperatures listed above in temperature-controlled water baths. The cells were then methanol-fixed and immunostained using anti-RSV-F mAbs as described in Materials and Methods.

### The missense P mutations increased Min A fitness *in vitro*

**Fig 2:**
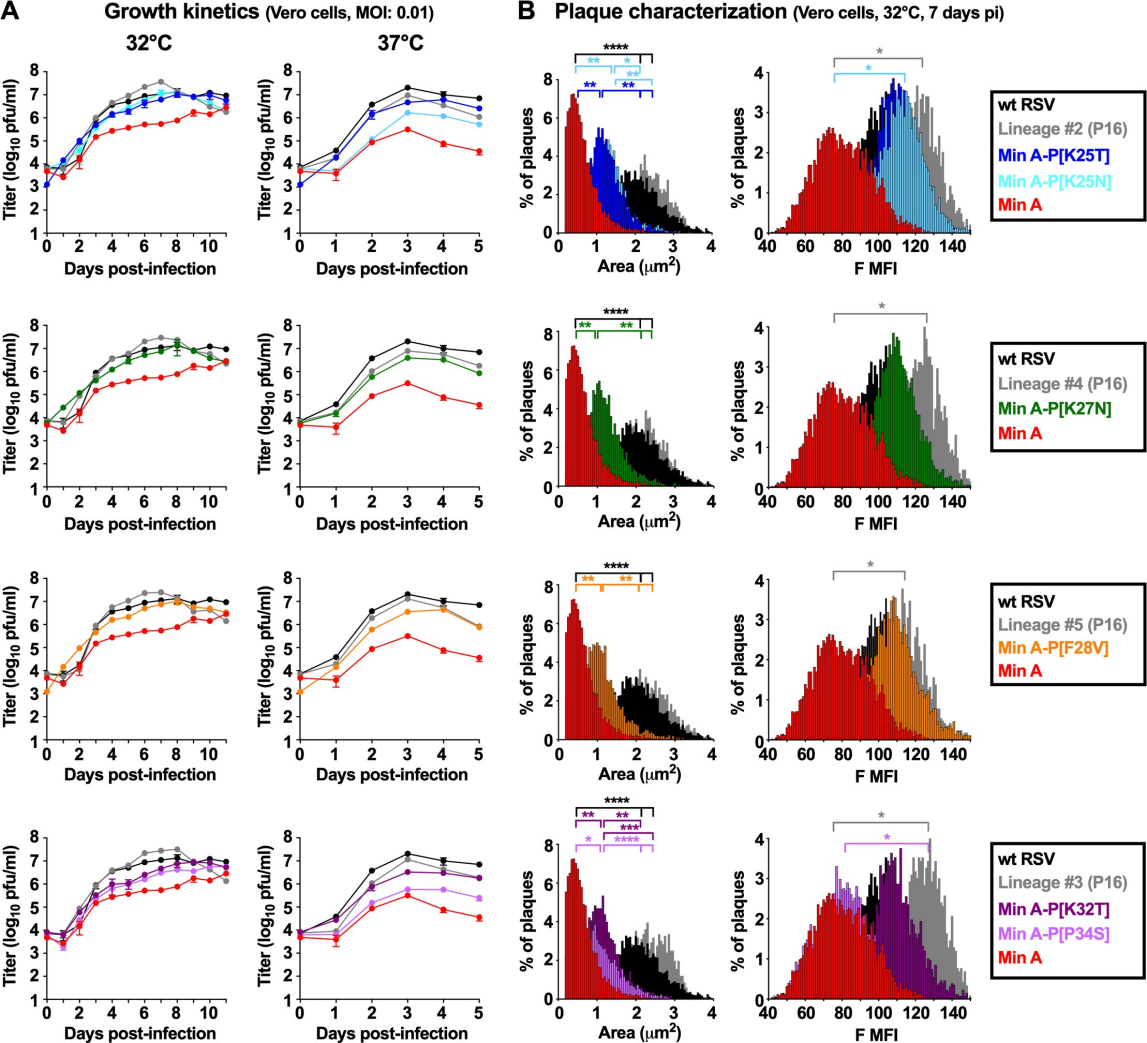
Effects of individual prominent P mutations on Min A replication, plaque size, and expression of F protein *in vitro*. **(A) Multi-cycle replication kinetics:** Vero cells in six-well plates were infected at an of MOI of 0.01 PFU/cell with wt RSV; Min A; P16 supernatant from Lineages #2, 3, 4, and 5 of the *in vitro* stress test; or Min A containing the individual indicated P mutations, and incubated at 32°C (left column) or 37°C (right column). For each virus, duplicate wells were harvested daily by scraping cells into the media followed by vortexing for 30 sec to release cell-associated virus. Clarified supernatants were snap-frozen until titrated by immunoplaque assay at the permissive temperature of 32°C. Titers for Min A, Min A-derived mutants, and wt RSV correspond to the mean of two replicate titrations each of two replicate wells at each timepoint. Due to limited sample size, titers of the P16 viruses correspond to the mean of two replicate titrations of one well at each time point. Day 0 titers correspond to the back titration of the inocula. Note that all viruses were evaluated side-by-side in a single experiment but for clarity are shown in four separate graphs for each temperature, with the specific viruses evaluated in each graph identified in the black boxes at the far right-hand side of Part B, with wt RSV (black) and Min A (red) being repeated in each graph. **(B) Plaque characterization**: Vero cells in six-well plates were incubated with 250 PFU/well of the indicated virus at 32°C. At day seven pi, plates were fixed with 80% methanol and plaques were stained with a cocktail of three anti-RSV F mAbs and a PE-labeled secondary antibodies as described in Materials and Methods. The plaque area (in μm^2^) and the level of RSV F expression (expressed as median fluorescence intensity, MFI) were evaluated on an average of 2140 (±1167) plaques per virus. Distributions of the virus plaque area and F MFI were compared for statistical significance using the ANOVA test (* = p≤0.05, ** = p≤0.01, *** = p≤0.001, **** = p≤0.0001).

We investigated the effects of the single P mutations on multicycle replication of Min A *in vitro* (Fig 2A). Vero cells were infected in duplicate at an MOI of 0.01 PFU/cell with: Min A; the indicated Min A-derived viruses bearing single P mutations; P16 of lineages #2, #3, #4, or #5; or wt RSV. P16 lineages were included in this set of experiments because of the limited material from the P18 lineages and the high virus titers obtained at P16. The presence in P16 of the prominent P mutations that we had originally identified in the P18 lineages was confirmed by Sanger sequencing, which showed that the appropriate mutations were present and predominant. Cells were incubated at the permissive temperature of 32°C (Fig 2A, left column) or the physiological temperature of 37°C (right column).

At 32°C, wt RSV replication peaked at 10^7.1^ PFU/ml on day seven post-infection (pi), as typically observed (Fig 2A, left column). As expected, Min A replication was reduced by about 10-fold compared to wt RSV (10^5.9^ PFU/ml at day 7) and reached a maximal titer only at day 11 (10^6.5^ PFU/ml). All of the Min A-derived mutants replicated more efficiently than Min A at 32°C; the P mutations at aa positions 25, 27, 28 and 32 conferred increases in Min A replication of up to 10-fold to peak titers of 10^6.8^–10^7.2^ PFU/ml, comparable to wt virus. The P mutation [P34S] was the least effective in increasing Min A replication (Fig 2A, bottom left; peak titer of 10^6.6^ PFU/ml); this mutation also had been the least effective in relieving temperature sensitivity, as noted above. For comparison, the replication of P16 from lineages #2 (containing the prominent mutation P[K25T]), #4 (containing P[K27N], #5 (containing P[F28V], and #3 (containing P[K32T] and P[P34S]) reached maximum titers of 10^7.4^–10^7.6^ PFU/ml at days seven or eight.

Comparable results were obtained at 37°C (Fig 2A, right column), except that maximum virus titers were reached at day three-four pi instead of day seven-eight pi that was observed at 32°C. The peak titer for wt RSV was 10^7.3^ PFU/ml, as typically observed. Min A replication at 37°C was approximately 60-fold reduced compared to wt RSV (peak titers of 10^5.5^ PFU/ml), consistent with previous results [20]. The presence of each of the P mutations at aa position 25, 27, 28 and 32 increased replication of Min A by about five- to 15-fold to peak titers of 10^6.2^–10^6.7^ PFU/ml, but these remained about four- to 12-fold lower than wt RSV. The mutation P[P34S], which had the least effect on temperature sensitivity and replication at 32°C, increased Min A replication at 37°C by only two-fold. Replication of P16 of lineages #2, #4, #5 and #3 also was increased compared to Min A, and reached 10^6.9^–10^7.1^ PFU/ml. Thus, the P mutations conferred increased replication at 32°C and 37°C but did not fully restore the level of replication at 37°C to that observed for wt RSV.

We also evaluated the effect of the P mutations on Min A fitness by characterizing the plaque sizes and the level of RSV F expression of individual plaques on Vero cells at 32°C (Fig 2B). Min A virus and wt RSV were included as controls, as well as P16 supernatants from lineages #2, #4, #5 and #3 (Fig 2B). Plaque sizes of all Min A-derived P mutants were significantly increased compared to Min A but remained intermediate between Min A and wt RSV. In contrast, the plaque sizes of the P16 virus stocks equaled or slightly exceeded that of wt RSV. With regard to the magnitude of expression of RSV F, all of the P mutations except for [P34S] increased the expression of RSV F per plaque, although not to the level of the P16 stocks or wt RSV. The increase compared to Min A was statistically significant for mutation P[K25N]. These data further confirmed that the individual P mutations improved Min A fitness, although not to the level of P16 stocks nor wt RSV.

### The missense P mutations restored Min A gene transcription to wt levels

We next investigated the effect of P mutations [K25T], [K27N], [F28V] and [K32T] on Min A RNA synthesis. These four mutations were the ones that had the greatest effect on increasing multicycle Min A replication at 37°C in the preceding section. Vero cells were infected at 37°C with an MOI of three PFU/cell of wt RSV, Min A, or the Min A-derived viruses bearing the individual missense mutations. Replicate samples were collected in four h-intervals from four to 24 hours (h) to monitor viral gene expression, protein expression, RNA replication, and virus replication in a single-cycle infection experiment.

**Fig 3:**
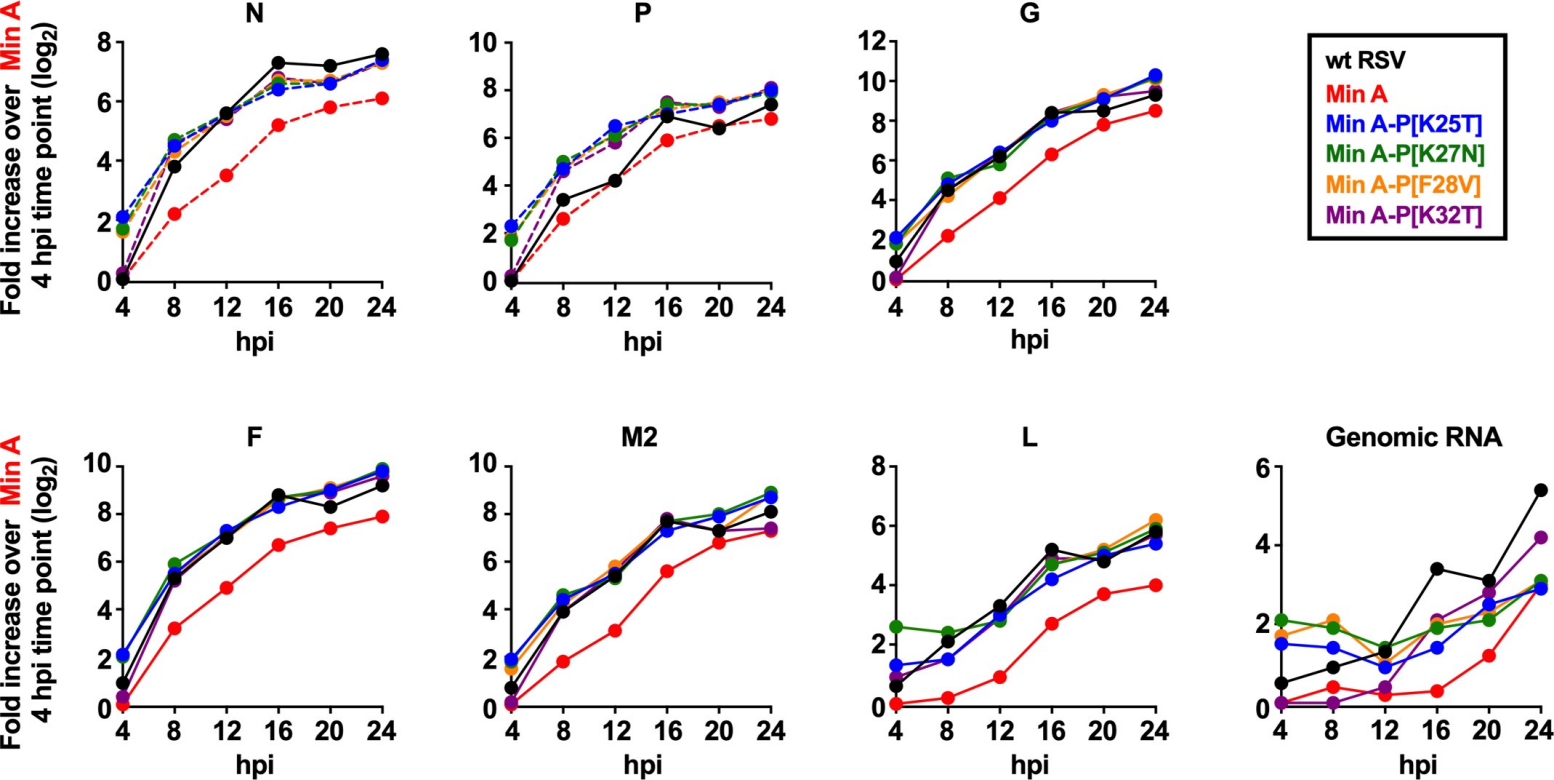
The P mutations restored RNA synthesis by Min A during a single-cycle infection. Replicate cultures of Vero cells were infected at an MOI of three PFU/cell with the indicated viruses at 37°C. Cell-associated RNA was collected every four h from four hpi to 24 hpi. Positive-sense RSV RNAs (mRNAs and antigenome) were quantified in triplicate by strand-specific RT-qPCR with tagged primers. Note that the CPD ORFs were sufficiently different in sequence from wt that they required the use of separate primers and probes. Data specific to the wt and CPD ORFs are shown with solid and dashed lines, respectively. Thus, expression of the CPD N and P ORFs of the Min A-derived viruses shown here cannot be directly compared to the N and P ORFs of wt RSV, whereas the other ORFs shown here [G, F, M2-1, and L] were wt in all viruses and thus can be compared directly between Min A-derived viruses and wt RSV. Negative-sense (i.e., genomic) RNA was detected by strand-specific RT-qPCR with tagged primers and a probe specific to the M2-1 ORF (solid lines). Data were normalized to 18S ribosomal (r)RNA and expressed as fold increase over Min A at the four hpi time point with the exception of N and P of wt RSV, which were expressed as fold-increase over wt RSV at the four hpi time point. Data for the remaining CPD genes (NS1, NS2, M, and SH) are shown in S1 Fig.

The levels of transcription and RNA replication were evaluated by RT-qPCR assays using tagged primers to separately quantify positive-sense RNA (which consists of mRNA and antigenomic RNA that typically are at a ratio of approximately 10:1 at the peak of RNA synthesis) and negative-sense genomic RNA (Figs 3 and S1). Positive-sense RNA was quantified with primers and probes for each of the viral ORFs except M2-2, whereas negative-sense RNA was quantified with primers and probe for the complementary strand of the M2-1 ORF. Fig 3 shows quantification of mRNA and antigenome for the N, P, G, F, M2 and L ORFs, as well as genome. S1 Fig shows quantification of mRNA and antigenome for the NS1, NS2, M and SH ORFs. Note that the quantification of the NS1, NS2, N, P, M and SH genes required different Taqman assays for Min A-derived viruses versus wt RSV because these ORFs were CPD in Min A-derivatives and wt in wt RSV. This precluded direct comparison of the Min A-derived viruses to wt RSV using these ORFs, although they are presented together in Figs 3 and S1 (wt ORFs are indicated by solid lines and CPD ORFs by dashed lines). Conversely, the G, F, M2-1, and L ORFs were wt in all viruses and could be directly compared (Fig 3).

Synthesis of positive-sense viral RNA, reflecting mainly mRNA, increased globally for all viruses from four to 24 hpi (Figs 3 and S1). When Min A and wt RSV were compared using the G, F, M2-1, and L ORFs that were identical in both viruses, the level of positive-sense RNA synthesis of wt RSV was about three- to eight-fold greater than that of Min A at all time points, confirming our previous results [20]. The insertion of each of the four P mutations increased the global positive-sense RNA levels of Min A (Figs 3 and S1) to that observed for wt RSV, suggesting that each of these P mutations completely restored viral transcription of Min A. In case of the NS1, NS2, N, P, M and SH ORFs, although direct comparison to wt RSV was not possible, the magnitude of the increase compared to Min A was comparable to that observed for the G, F, M2, and L mRNAs (Figs 3 and S1). Quantification of genomic RNA synthesis using tagged RT-qPCR specific for negative-strand RNA showed that the P mutations also increased the genomic RNA synthesis of Min A two to four-fold between 16 and 20 hpi.

### The missense P mutations increased Min A protein expression and virus replication

We next investigated the level of cell-associated viral protein expression as well as virus replication in Vero cells from the same single-cycle infection experiment (MOI of three PFU/cell, 37°C) that was described in Fig 3. Replicate cultures of infected cells were harvested for analysis at four-h intervals from four to 24 hpi.

**Fig 4:**
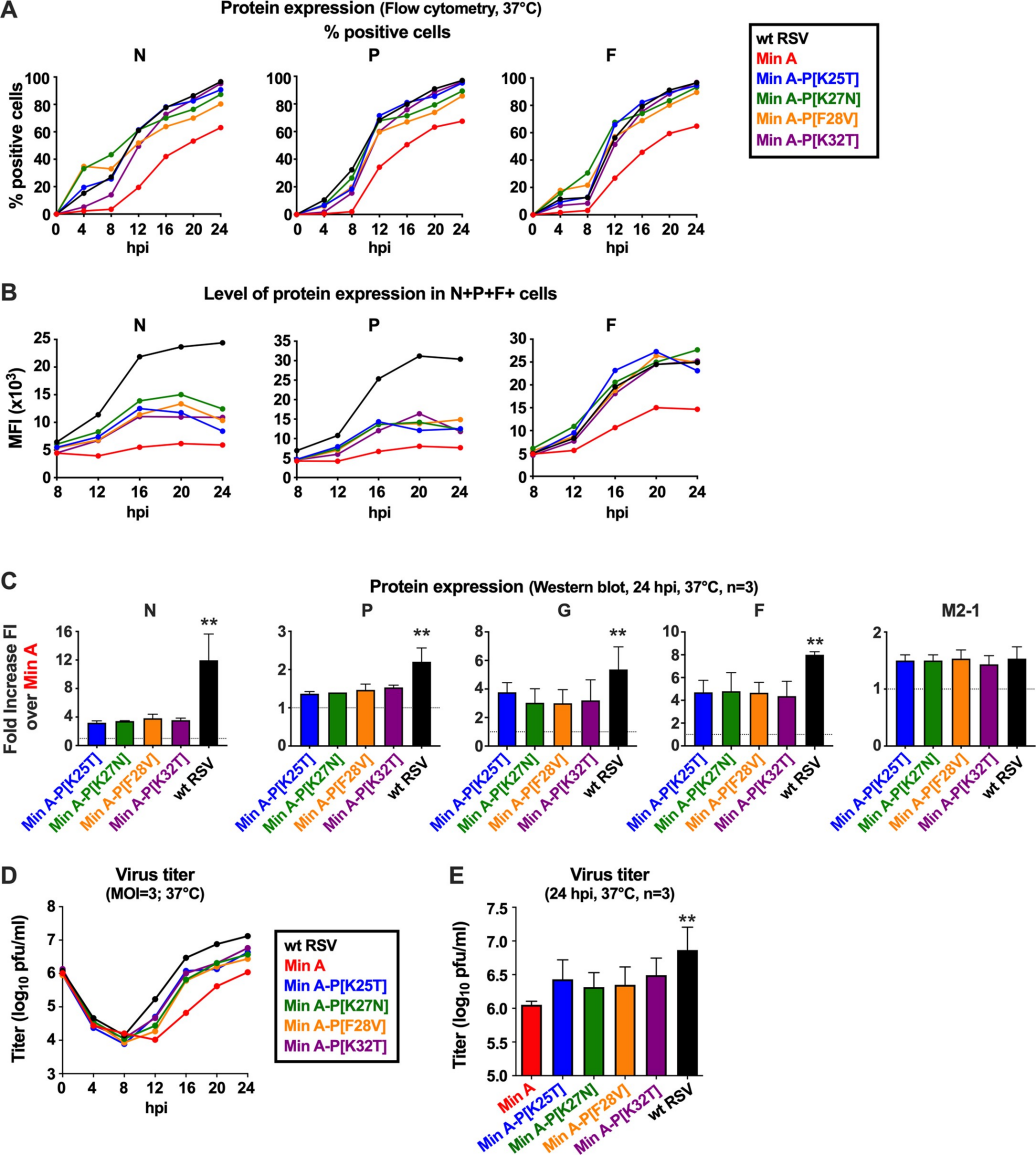
The P mutations increased Min A protein expression and virion production during a single-cycle infection. **(A, B, C) Protein expression**. (A, B) Additional replicate Vero cell monolayers in six-well dishes from the single-cycle infection experiment in Fig 3 (MOI of three PFU/cell, 37°C) were harvested at four-h intervals from four to 24 hpi (one well per virus per time point) for analysis of protein expression by flow cytometry (A, B). (A, B) Cells were harvested, fixed, permeabilized, immunostained, and subjected to flow cytometry to determine the percentage of cells that expressed N, P, and F proteins (A) as well as the median fluorescence intensity (MFI) of the positive cells (B). (C) Western blotting from additional replicate Vero cell monolayers in six-well dishes from the single-cycle infection experiment in Fig 3 and from two additional independent repeat experiments of the 24-h time point were performed. The Western blot data were combined (n = 3) and expressed as the mean fold-increase in fluorescence intensity (FI) over Min A, with the standard deviation shown. **(D-E) Virus production.** (D) Additional replicate Vero cell monolayers in six-well dishes from the experiment in Fig 3 (MOI of three PFU/cell, 37°C) were harvested at four-h intervals from four to 24 hpi (one well per virus per time point) for analysis of virus production by immunoplaque assay of clarified cell-culture-medium supernatants. (E) Two additional independent repeat experiments of the 24-h time point in panel D were performed, and the data from these two experiments were combined with those from D and are expressed as mean values with the standard deviation shown. In panels C and E, statistical differences indicated at the top of each graph are in comparison to Min A (**p ≤ 0.01).

Viral protein expression was analyzed by flow cytometry (Fig 4A and 4B) and Western blotting (Fig 4C). Flow cytometry analysis showed that, for all of the mutant and control viruses, the percentage of cells that were positive for the N, P or F proteins increased steadily from eight to 24 hpi (Fig 4A). However, Min A infection seemed to progress at a slower rate: at 20 to 24 hpi, the percentage N-, P- or F-positive cells was about two-fold lower than with wt RSV-infected cultures. In comparison, the percentage of positive cells for the Min A-derived viruses with P mutations was in the same range to that of wt RSV.

In addition, we investigated the level of expression (expressed as median fluorescence intensity, MFI) of N, P, and F protein in cells co-expressing all three proteins (Fig 4B). We found that the level of protein expression from the CPD N and P ORFs and the non-CPD F ORF in Min A derivatives containing the individual P mutations was increased by about two-fold compared to Min A. However, expression of N and P protein by the Min A-derived viruses with P mutations was still about two- to three-fold lower than for wt RSV, whereas the level of expression of F protein was restored to that of wt RSV.

Thus, the individual P mutations restored mRNA transcription by Min A mutants to levels similar to wt RSV (as shown in Fig 3), which also restored the level of expression of the non-CPD F protein to that of wt RSV. In contrast, expression of N and P proteins from the CPD ORFs remained reduced compared to wt RSV. This would be consistent with the paradigm that CPD reduces the efficiency of translation.

Additional replicate cultures were analyzed by Western blotting with antibodies specific to the G, F, N, P, and M2-1 proteins. Two additional repeats of the single-cycle infection experiment were performed in which infected cells were harvested at 24 hpi and subjected to the same Western blot analysis. These data were quantified together and are shown in Fig 4C, expressed as fold-increase over Min A at 24 hpi. These results confirmed that the introduction of each of the P mutations into Min A increased the expression of the G, F, N, P, and M2-1 proteins. Specifically, the expression of N, G and F was increased by three- to five-fold compared to Min A, and the expression of P and M2-1 was modestly increased by 1.3- to 1.5-fold. However, except for M2-1, the level of viral protein expression by the Min A-derived viruses containing individual P mutations remained lower compared to wt RSV. The finding that expression of G and F from non-CPD ORFs was not restored to wt levels when measured by Western blot (Fig 4C) whereas expression of F was restored when measured by flow cytometry (Fig 4B) might be a consequence of differences in how the two assays are affected by the reduced efficiency of infection by the Min A-derived viruses compared to wt RSV. The MFI determined by flow cytometry is based only on infected cells and thus can be compared between cultures with different percentages of infected cells. In contrast, Western blotting values are directly affected by differences in the percentages of infected cells between cultures. Thus, MFI determined by flow cytometry probably is the more relevant comparison for efficiency of expression per infected cell.

We also evaluated the kinetics of virus replication from the same single-cycle infection experiment described above. Thus, replicate infected Vero cell cultures (MOI three PFU/cell, 37°C) were harvested at four-h intervals from four to 24 hpi, and clarified cell-culture-medium supernatants were prepared and analyzed by immunoplaque assay to quantify infectious virus titers (Fig 4D). Progeny viruses were first detected at approximately 12 hpi. At 24 hpi, wt RSV titers reached 10^7^ PFU/ml, as typically observed. Min A replication was about 10-fold lower compared to wt RSV, whereas replication of the Min A derivatives bearing individual P mutations was approximately five-fold higher than Min A but less than wt RSV (Fig 4D). In addition, two additional repeats of the single-cycle infection experiment were performed in which infected cells were harvested at 24 hpi, and clarified culture medium supernatants were prepared and analyzed by immunoplaque assay; data were combined with that from Fig 4D to create Fig 4E. This confirmed that the individual P mutations increased Min A replication by about five-fold, but not to the level of wt RSV.

Taken together, the levels of positive-sense RNAs in single-cycle replication experiments (Fig 3) suggested that the P mutations restored Min A gene transcription to wt RSV level. However, quantification of protein expression (Fig 4) suggested that the translation of the CPD mRNAs remained reduced compared to wt mRNAs, thus resulting in reduced protein expression and reduced virus replication compared to wt RSV.

### Replication, immunogenicity, and protective efficacy in hamsters of Min A-derived viruses bearing individual P mutations

We evaluated the effects of the individual P mutations on Min A replication, immunogenicity, and protective efficacy in hamsters. Fourteen hamsters per group were inoculated intranasally with 10^6^ PFU of wt RSV, Min A or the Min A-derived viruses P[K25T], [K27N], [K28V], and [K32T], and an additional three animals per group were included as unvaccinated controls.

**Fig 5:**
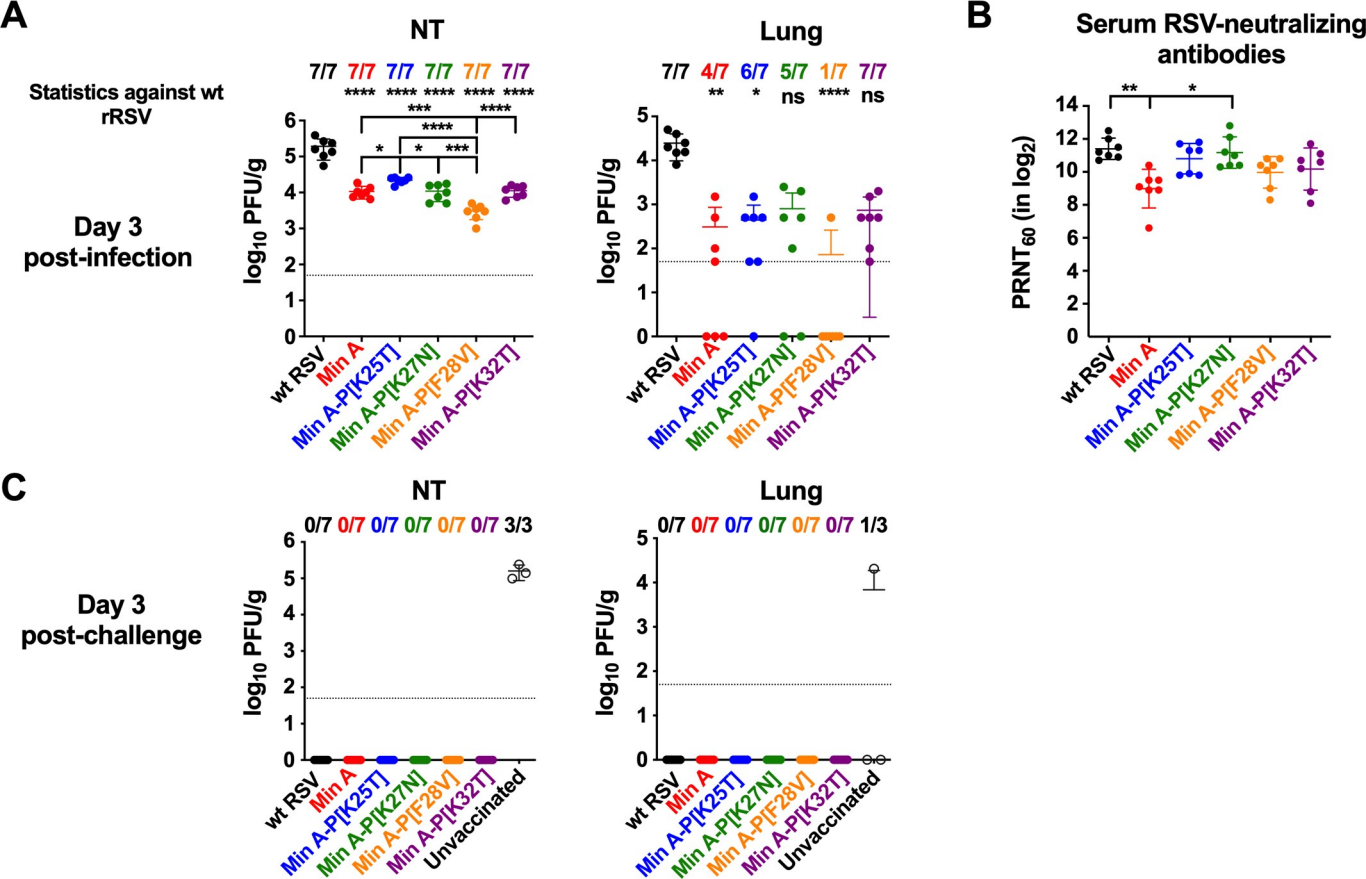
Replication, immunogenicity, and protective efficacy of the Min A-derived viruses in hamsters. Groups of 14 six-week old golden Syrian hamsters were inoculated intranasally with 10^6^ PFU of the indicated virus per animal. Three additional hamsters were left uninfected as controls. (A) Replication of wt RSV, Min A and Min A-derivatives in the nasal turbinates (NT) and lungs harvested at day three pi from seven hamsters per group was evaluated by plaque assay as described in Materials and Methods and expressed as PFU/g of tissue. The limit of detection, 50 PFU/g, is indicated by a dotted line. (B) Sera were collected at day 24 pi from seven hamsters per group and the 60% plaque reduction neutralizing antibody titers (PRNT_60_) were determined. (C) At day 27 pi, seven hamsters per group and the three control hamsters were challenged by intranasal inoculation with 10^6^ PFU of wt RSV. Three days after challenge, replication of wt RSV was evaluated in the NT and lungs by plaque assay. In each graph each hamster is represented by a colored circle and the median value and standard deviation are shown. The number of hamsters with replicating virus is indicated. In panel A, statistical differences indicated at the top of each graph are in comparison to wt RSV while differences between Min A and Min A derivatives are indicated in brackets. In panel B, all statistical differences are indicated by brackets (*p ≤ 0.05; **p ≤ 0.01; ***p ≤ 0.001; ****p ≤ 0.0001).

Virus replication was evaluated at day three pi: seven animals per group were euthanized, nasal turbinates (NT) and lungs were harvested and homogenized, and viral titers were quantified by immunoplaque assay (Fig 5A). In the NT, Min A replication was significantly reduced (by about 18-fold) compared to wt RSV (p≤0.0001) which replicated to about 10^5^ PFU/g, as typically observed. Interestingly, introduction of the P mutations did not cause de-attenuation in the NT except in the case of P[K25T] which induced a partial de-attenuation. Specifically, introduction of the P[K27N] or P[K32T] mutations into Min A did not affect its replication in the NT; mutation P[F28V] reduced rather than increased Min A replication by about four-fold (p≤0.001); and P[K25T] mutation induced a modest but significant increase of Min A replication in the NT (p≤0.05).

In the lungs, Min A replication was strongly reduced compared to wt RSV (p≤0.01). Indeed, Min A was detected in only four out of seven hamsters and at 10-to-100-fold lower levels than in wt RSV. None of the P mutations caused de-attenuation in the lungs: mutations P[K25T], P[K27N] and P[K32T] did not have a significant effect on Min A replication, and mutation P[F28V] appeared to reduce Min A replication in the lungs (as it did in the NT), as only one out of seven hamsters exhibited virus replication.

On day 24, sera were collected from the remaining seven virus-infected animals per group and analyzed by a complement-enhanced 60% plaque reduction neutralization assay (PRNT_60_). Min A and the Min A-derivatives induced high levels of serum RSV-neutralizing antibodies (Fig 5B). However, the Min A antibody response was significantly lower than that induced by wt RSV (p≤0.01). Introduction of the individual P mutations induced a modest increase to levels that were not statistically different than wt RSV. Introduction of the P[K27N] mutation in particular induced a significant increase of neutralizing antibodies compared to Min A (p≤0.05).

On day 27 pi, the remaining seven virus-infected hamsters and three uninfected controls per group were challenged intranasally with 10^6^ PFU of wt RSV. The animals were euthanized on day three post-challenge, and replication of challenge virus was measured in homogenates of the NT and lungs. As expected, unvaccinated hamsters had substantial titers of challenge virus in the NT (around 10^5^ PFU/g), and one of three hamsters had virus in the lungs (10^4.3^ PFU/g). However, no challenge wt RSV was detected in the NT or lung of any of the vaccinated hamsters, showing that these animals were fully protected against wt RSV.

Thus, we identified two viruses of particular interest; Min A-P[K27N] and Min A-P[F28V]. Min A-P[K27N] replicated to a similar level as Min A in both the NT and lungs, but induced titers of serum neutralizing antibodies that were significantly higher than for Min A and equivalent to wt RSV. Replication of Min A-P[F28V] was significantly reduced compared to Min A (NT) and wt RSV (NT and lung), but this virus induced an antibody response that was not significantly different than wt RSV. Thus, these two viruses warrant further investigation as improved Min A-derived vaccine candidates.

### Genetic stability of the Min A-P[K27N] and Min A-P[F28V] viruses

The genetic stability of the Min A-P[K27N] and Min A-P[F28V] viruses was evaluated in a temperature stress test involving four passages at 39°C and four passages at 40°C, corresponding to two months of continuous culture (S2 Fig). Each virus was also passaged the same number of times in parallel at the permissive temperature of 32°C as control. Sanger sequencing of the complete genomes of the three different stressed replicates and the two control replicates was performed at the end of the final passage (P8).

In the case of Min A-P[K27N], no prominent mutations were found at P8 in two of the three stressed replicates nor in the two control replicates. The remaining stressed replicate contained three subdominant mutations in L ([N1473D], [N1475D] and [I14771R]). Thus, Min A-P[K27N] exhibited substantially increased genetic stability.

In the case of Min A-P[F28V], no prominent mutations were found in two of three stressed replicates nor in one control replicate. The remaining control lineage contained one prominent mutation in the 3’UTR of F and four prominent missense mutations in M2-1: ([H14R], [N40S], [M50V] and [K52R]). These same four mutations in M2-1 were present in the remaining stressed lineage, but each was subdominant. These four missense mutations in M2-1 were “t” to “c” mutations, suggesting that they were introduced on the genomic RNA by cellular deaminases.

While Min A-P[F28V] still appeared to exhibit some residual level of instability in one lineage, the pattern was consistent with cytidine deaminase activity rather than polymerase infidelity [34]. Unlike for the parental virus Min A, no clear pattern of prominent instability emerged during the temperature stress test, showing that the introduction of these P mutations led to substantial improvements in stability.

### Effects of additional prominent mutations that co-emerged with P[K27N] and P[F28V] during the serial passages of Min A

Several additional prominent mutations that had co-emerged with P[K27N] (in lineage #4) and P[F28V] (lineage #5) during the serial passages of Min A (Table 1) were re-introduced into Min A-P[K27N] and Min A-P[F28V]. Specifically, in addition to P[K27N], lineage #4 had accumulated four other prominent mutations (Table 1): one was in the 5’ UTR of the N gene, specifically a1138g in the Kozak sequence [35] at position -3 preceding the translation initiation codon; a second was the missense mutation [V151A] in the L ORF; the remaining two were silent changes in the N and P ORFs and were predicted to be inconsequential and were not examined further. Mutations a1138g and L[V151A] were introduced into the Min A-P[K27N] backbone to generate the virus Min A-P[K27N]+2 (S3 Fig).

In addition to P[F28V], lineage #5 had accumulated five other prominent mutations (Table 1): two were missense in M ([K123M]) and L ([S2084P]), and three were non-coding, occurring in the NS2 5’UTR (t612c), the P gene-start signal (c2334t, GGGGCAAAT) as already noted, and the P 3’UTR (a3195g). We re-introduced these five mutations into Min A-P[F28V] backbone by reverse genetics in two combinations: (i) the two missense mutations in M ([K123M]) and L ([S2084P]) in addition to the P[F28V] mutation, resulting in the virus Min A-P[F28V]+2, and (ii) all five prominent mutations in addition to the P[F28V] mutation, resulting in Min A-P[F28V]+5 (S3 Fig). The sequence of each virus was confirmed by Sanger sequencing.

Evaluation of these viruses in a multicycle replication experiment in Vero cells incubated at 32°C or 37°C showed that the introduction of these additional mutations into Min A-P[K27N] and Min A-P[F28V] did not further improve the replication of these viruses (S3 Fig). This further confirmed the critical role of the original P[K27N] and P[F28V] mutations in individually conferring increased Min A replication. In addition, the presence of the additional missense mutations did not significantly alter the replication and immunogenicity of the Min A-derived P mutants in hamsters (S4 Fig), except for Min A-P[F28V]+5, which exhibited slightly reduced replication and immunogenicity compared to Min A-P[F28V].

**Fig 6:**
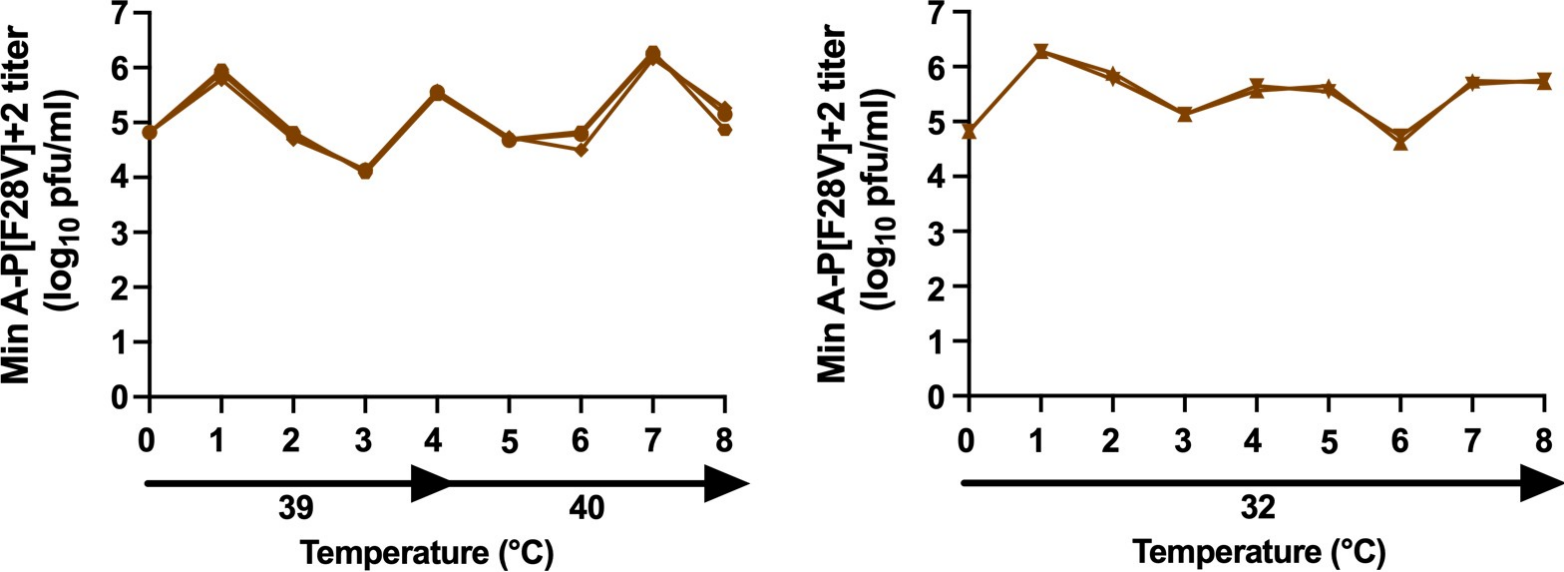
Min A-P[F28V]+2 was phenotypically stable in a temperature stress test. The stability of Min A-P[F28V]+2 that contains the M[K123M] and L[S2084P] mutations in addition of the mutation P[F28V] (see S3 Fig) was evaluated in a temperature stress test. Five replicate cultures of Vero cells in 25 cm^2^ flasks were inoculated with an MOI of 0.1 PFU/cell. Three replicate cultures (left panel) were incubated at 39°C for four passages and 40°C for an additional four passages, representing two months of culture. The remaining two replicate cultures (right panel) were passaged in parallel for eight passages at the permissive temperature of 32°C. Flasks were harvested when extensive syncytia were observed or when the cells started to detach. Clarified fluids from the previous passage were used to infect the following passage of fresh cells in a 1:5 dilution. In addition, aliquots of clarified virus from each lineage were snap frozen for virus titration by plaque assay at 32°C. Whole genome Sanger sequencing was performed at the end of the experiment (P8).

We evaluated the genetic stability of Min A-P[F28V]+2 in a temperature stress test involving four passages at 39°C and four passages at 40°C, corresponding to two months of continuous passage (Fig 6). This virus was also subjected to eight passages in parallel at the permissive temperature of 32°C as a control. No predominant mutations, and only three subdominant missense mutations, were found in the five lineages at the end of the stress test. Thus, Min A-P[F28V]+2 exhibited a further increased genetic stability compared to Min A and Min A-P[F28V] and represents an improved Min A-derived vaccine candidate for RSV.

### Computational analysis of the effects of P[K27N] and P[F28V] on the N-terminal region of P and on the N-P complex

**Fig 7:**
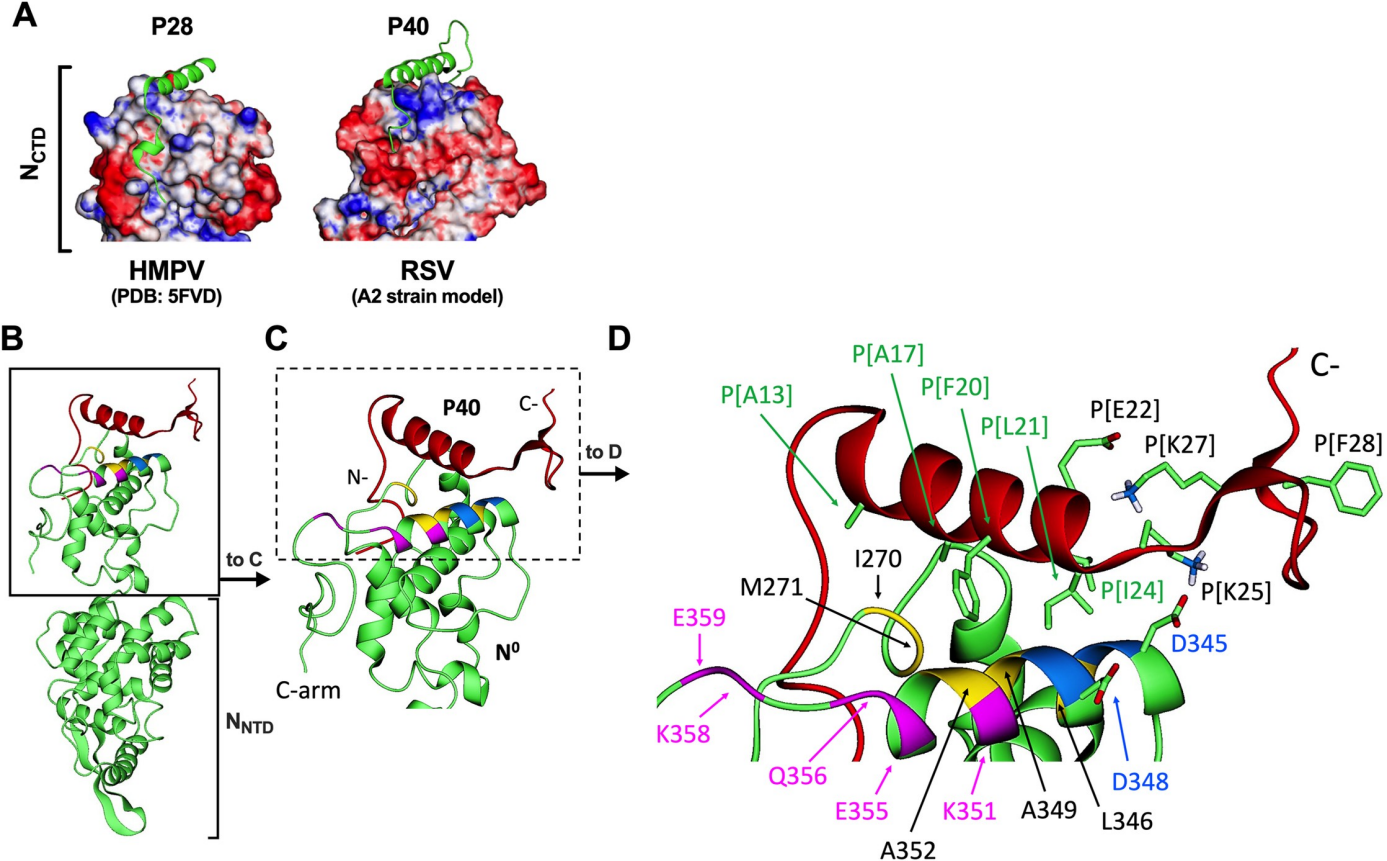
Molecular dynamic (MD) simulations of the effects of P mutations K27N and F28V on interactions within a complex of a peptide representing the N-terminal 40 aa of the RSV P protein (P40) with monomeric N protein (N^0^) in solution. (A) Comparison of (left) the published [36] crystal structure (PDB 5FVD) of HMPV N^0^ protein complexed with a peptide containing aa 1–28 of the HMPV P protein (P28), with (right) our model of wt RSV N^0^-P40 structure obtained from MD simulations. The C-terminal domains (CTD) of N^0^ of HMPV and RSV are shown. HMPV P28 and RSV P40 are shown as green ribbons; the molecular surface electrostatic potentials of N^0^ are colored red (negative potential) and blue (positive). (B, C, D) Ribbon diagram models of the RSV N^0^ protein (green) complexed with wt P40 (red) generated from the MD simulations upon full equilibration (see Materials and Methods for details). Panel B shows the complete N^0^ protein complexed with P40: the NTD (aa 31–249) of the N^0^ protein is indicated with a bracket at the bottom (the N-term arm aa 1–30 is removed from full-length N to create the soluble N^0^), and the CTD (aa 256–359) and C-term arm (aa 360–391), together with the P40 peptide (red), are boxed at the top with a solid line and shown in an expanded view in C. In panel C, P40 and the interface with N^0^ CTD are boxed with a dotted line and shown in an expanded view in D. In panel D, key residues (see discussion in the text) are identified by sequence position: P40 residues are labeled as P[]; all other residues are in N^0^. In some cases, aa side chains are shown. In panels C and D, color-coding was used to indicate critical residues of N^0^ involved in persistent H-bond/salt-bridge and hydrophobic/nonpolar interactions with P40: (i) blue indicates N^0^ residues [D345] and [D348] interacting frequently with [K25] of P40; (ii) yellow indicates hydrophobic N^0^ residues I270, M271, L346, A349, A352 involved in persistent interactions with several nonpolar residues of P40 (A13, A17, F20, L21 and I24) thus providing a bed of nonspecific hydrophobic interactions upon which the P40 helix rests; and (iii) purple indicates N^0^ residues E355, K351, E359, Q356, K358 (ranked in decreasing order of importance) involved in interactions with the P40 mutants but not with the wt P40 that is shown in this figure.

Previous structural studies of RSV and the related pneumovirus human metapneumovirus (HMPV) showed that the N-terminal region of the P protein interacts with the C-terminal domain (CTD) of monomeric RNA-free N (N^0^) and serves as a chaperone that maintains N^0^ in its monomeric state [27,36]. The crystal structure of HMPV N^0^ in complex with a peptide representing aa 1–28 of the P protein (P28) was previously solved ([36], Protein Data Bank (PDB) 5FVD; Fig 7A, left panel). As shown in Fig 7A, the HMPV P28 peptide (green ribbon) is stabilized in the complex by hydrophobic and electrostatic interactions with surface residues of the N^0^ CTD; positive and negative potentials on the surface of N^0^ are colored blue and red, respectively. Residues 12–28 of P28 adopt an α-helical structure (Fig 7A, left) [28,36].

For RSV, only low-resolution models of a complex between a truncated soluble form of the RSV N protein and a peptide representing the N-terminal end of the RSV P protein (P40; aa 1–40) were available [37]. These models similarly predict RSV P40 interacting with part of the RSV N^0^ CTD (aa 256–359) and residues 10–24 of RSV P40 having an α-helical structure [37]. These models of N^0^-P40 interactions had been determined using N and P40 sequences bearing deletions and/or amino acid substitutions designed to facilitate computation [37].

We used the model based on the HMPV atomic structure (PDB 5FVD), together with complete authentic sequences of RSV N and P40, to generate an *in silico* atomic-resolution model of the RSV N^0^-P40 complex through molecular dynamics (MD) simulations (Fig 7A, right panel). Our model suggests that hydrophobic and electrostatic interactions also play a role in stabilizing P40 on the N_CTD_ domain of RSV and predicts close similarities between the HMPV and RSV complexes.

We then subjected the atomic-resolution model of the RSV N^0^-P40 complex to comparative MD simulations to gain insight into the effects of the P[K27N] and P[F28V] mutations on the N^0^-P40 complex (Fig 7B, 7C and 7D, showing N^0^ in green and P40 in red). First, we evaluated interactions within P40 while it was complexed with the N^0^ CTD. This modeling suggested that the wt assignment P[K27] forms hydrogen bonds with several acidic residues of P40, notably E22, side chain shown in Fig 7D, and other residues located on both sides of residue 27 (e.g., S23, T29, and D33, Fig 7D, side chains and aa assignments not shown to preserve the clarity of the ribbon diagrams). When the mutant assignment of 27N was introduced, our modeling suggested that this assignment may be less effective than the original wt assignment of K in forming hydrogen bonds with these acidic P40 residues nearby. A reduced ability to form intra-P40 hydrogen bonds could render P40 bearing P[K27N] more flexible. In the case of residue 28, the aromatic side chain of the wt assignment F28 (Fig 7D) appeared to provide wt P40 with a more compact local arrangement of several nonpolar residues of P40, especially P31 and the hydrocarbon chains of several lysine residues nearby, e.g., K25, and K37 (Fig 7D; side chains and aa assignments not shown for P31 and K37). When the mutant assignment of V28 was introduced, it made this nonpolar packing less effective, resulting in an increase in the segment flexibility. Thus, the P[K27N] and P[F28V] mutants are predicted to confer, by different mechanisms, increased flexibility to the disordered C-terminus of P40.

Next, we analyzed the interactions between P40 and N^0^ in the N^0^-P40 complex in both the wt (Fig 7D) and the mutants. This revealed critical interactions between P40 and N^0^. Many N^0^ residues interacted with P40 similarly in the wt and the mutants, suggesting fundamental interactions necessary to stabilize the complex. For example, through persistent H-bond or salt-bridge interactions, [D345] and [D348] (blue) of N^0^ frequently interacted with the wt assignment [K25] of P40 (Fig 7D). These interactions are essential in stabilizing the C-terminal end of the P40 helix. Likewise, hydrophobic residues of N^0^, notably I270, M271, L346, A349, A352 (shown in yellow, Fig 7C and 7D), showed persistent hydrophobic/nonpolar interactions with several nonpolar residues in the P40 helix (A13, A17, F20, L21 and I24, indicated in Fig 7D), thus providing a bed of nonspecific hydrophobic interactions upon which the P40 helix rests (Fig 7C and 7D).

Several interactions were unique to the P40 mutants [K27N] or [F28V] and not observed with wt P40. Specifically, our simulations predicted interactions of P40 containing [K27N] or [F28V] with polar or charged residues of the N^0^ CTD, including E355, K351, E359, Q356, K358 (shown in magenta in Fig 7D; ranked in decreasing order of importance, as assessed by the frequency of interactions during the dynamics). These residues are located at the end of a helix where the disordered C-arm of N^0^ begins. Our analysis of the frequency of H-bonds and hydrophobic contacts showed that these N^0^ residues interact with the disordered C-terminus of P40 mutants [K27N] or [F28V] but not with wt P40.

Thus, our computational study predicts that the P mutations may change the thermodynamics and/or the kinetics of N-P complex formation by increasing the flexibility of P, leading to better structural adaptation during P:N complexation. The mutations may impact nucleocapsid assembly and could potentially explain the increase in efficiency of the Min A transcription/replication complex.

## Discussion

Genome-scale deoptimization by synonymous recoding of ORFs of viral pathogens is a strategy to generate new types of live attenuated vaccine candidates that is gaining increasing use. The large number of nucleotide substitutions introduced into the genome is expected to confer increased genetic stability. This strategy is expected to reduce the possibility of de-attenuation, which is one of the main concerns for live-attenuated vaccines. However, this paradigm had not been rigorously tested. Therefore, prior to evaluating a genome-scale deoptimized vaccine candidate in clinical trials, an in-depth evaluation of genetic stability is important.

In the present study, we investigated the genetic stability of Min A, an RSV vaccine candidate in which the six promoter-proximal genes, NS1, NS2, N, P, M and SH, were subjected to CPD. Min A was moderately temperature sensitive. When Min A was subjected to serial passage at increasing temperatures, it lost much of its temperature sensitivity and restriction, presumably due to the acquisition of compensatory mutations. Full-genome deep sequencing showed that the nine stressed lineages and, somewhat surprisingly, the two control lineages had acquired a wide array of point mutations that involved every ORF, were mostly different between lineages, and had a high proportion of missense mutations. The P ORF was a frequent target: eight of the nine stressed Min A lineages acquired one or two prominent missense mutations in the N-terminal region (aa 25–32) of the P protein, which is a multifunctional protein that acts as a polymerase co-factor and a chaperone for soluble N protein, among other activities. When reintroduced into Min A, these P mutations individually induced a substantial reversion of the ts phenotype and a substantial reversion of the restriction of Min A replication at 32 and 37°C.

In single-cycle infection experiments, reintroduction of these P mutations into Min A restored the intracellular accumulation of viral positive-sense RNA (comprised mainly of mRNA) for each of the evaluated genes to wt levels and substantially restored the accumulation of progeny genomes. Thus, it was an effect on the accumulation of all of the positive-sense RNAs, rather than P alone, as well as on the viral genome. However, while the global level of Min A protein expression was increased by the P mutations, it did not reach wt levels for proteins from CPD ORFs. On a single-cell level, protein expression of the non-CPD F ORF by Min A versions with P mutations reached the same level as wt RSV, whereas protein expression of the CPD N and CPD P ORFs was increased but remained below that of wt RSV. This suggested that the Min A CPD transcripts were not as efficiently translated as wt transcripts, consistent with the idea that CPD reduces the efficiency of mRNA translation [2,4]. CPD can also affect mRNA stability, but this was probably not the cause of the reduced protein levels here because the levels of CPD positive-sense RNAs were restored to wt levels.

The increased transcription that we observed when the P mutations were introduced into Min A suggested that these missense mutations increased the efficiency of the polymerase complex to compensate for the reduced protein expression of the CPD mRNAs. The overall compensatory effects on transcription by the mutations identified in the N terminal region of the P ORF on Min A were very similar to those by mutations accumulating in the M2-1 ORF of another CPD vaccine candidate, Min L [21]. Thus, in two CPD versions of RSV (Min L and Min A), the increased efficacy of virus replication that occurred during selective pressure was due to the acquisition of missense mutations in proteins of the polymerase complex, with the most prominent effects observed for mutations in M2-1 [21], and in the phosphoprotein P (this study). As noted, the L polymerase also was a frequent target for mutations in the present study, but these were not evaluated in the present study except for the L[V151A] and L[S2084P] prominent mutations in lineages #4 and #5, respectively, which had little additional effect when reintroduced into Min A together with predominant P mutations at positions 27 and 28.

The RSV P protein forms tetramers and serves as a polymerase cofactor that interacts with the N, M2-1 and L proteins [12]. P serves as a chaperone protein to the newly-synthesized RNA-free N (N^0^) and is thought to stabilize the viral polymerase onto the nucleoprotein-RNA (N-RNA) complex [37,38]. RSV P also recruits the RSV transcription elongation factor M2-1 for efficient transcription of the viral RNA [37,38]. There are three functional domains described for RSV P; the P_NTD_ from aa one to ~120, the oligomerization domain (P_OD_) from aa 120 to 160, and the C terminus (P_CTD_) from aa 161 to 241 [39]. The structure of a short 40-mer peptide of the N-terminal domain of P (aa 1–40, P40) has been described in a complex with N^0^ [37]. Partial structures for the P_OD_ and P_CTD_ regions have been recently elucidated in complex with L. However, the structure of the P_NTD_ region beyond P40 (residues 41 to ~120) is still unknown [38,40]. The region of aa one to 99 is predicted to be disordered because it contains a high proportion of aa residues such as proline and lysine that promote disorder [41]. P40 is thought to adopt an α-helix spanning residues ~10–24, followed by a flexible region spanning the remainder of the peptide [37]. In addition, the P_NTD_ was found to interact with the monomeric form of N in solution [27].

We performed 3D molecular modeling of the potential effects of two prominent mutations in the P protein, namely K27N and F28V on the structure of RSV P40 in complex with N^0^. Our modeling was guided by a crystal structure of the HMPV N^0^ protein complexed with a peptide containing aa 1–28 of HMPV P protein (P28 peptide) [36,42], as well as low-resolution models of soluble RSV N protein complexed with the RSV P40 peptide [37,42]. In the models presented, RSV P40 binds to CTD of N^0^. In addition, the available low-resolution models of the complex between P40 and N^0^ had been determined experimentally complemented with *in silico* experimentation using P40 and N^0^ molecules that had been modified *in silico* to facilitate computation. Our computation analysis of the interactions of N^0^ and P40 of RSV was *in silico* and employed complete P40 and N^0^ that were entirely wt except for the single aa mutations at P40 positions 27 and 28. Our modeling showed that, when complexed with N^0^, the structural flexibility of the disordered aa 25–40 region of P40 was increased by either of the single aa mutations at positions 27 or 28. The increase in flexibility might be sufficient to allow structural adaptations during the N-P association/dissociation through changes in binding affinity or kinetics.

We presume that the major deleterious effect of CPD on the viral polymerase complex of Min A was indirect and due to the reduced synthesis of these polymerase-related proteins from CPD ORFs, namely N and P but not M2-1, M2-2, or L. The reduced expression of P and N by Min A was confirmed. We believe that the mutations accumulated during the initial stress test were amplified and maintained because they compensated for the effects of CPD. For example, the prominent P mutations may have compensated for the reduced quantity of polymerase complex components, especially P, by increasing the efficiency of the functions of P protein. This could involve the increased flexibility predicted from the modeling study. The same degree of flexibility could probably be attained with several other P mutations (S1 Table), whereas others may increase or decrease the flexibility beyond what is required or even induce stable secondary structures, hampering the replication process. Although the modeling was based on P40, the effects described in this study could possibly be present in full-length P, in which case the enhanced flexibility would be reflected in a more mobile N-terminal helix. In addition, the prominent mutations at residues 27 and 28 of P40 were predicted to result in new interactions with residues in N^0^. These interactions might increase the efficiency of binding of P to N^0^, which, in turn, could increase the efficiency of nucleocapsid-associated functions.

In the previous study evaluating the genetic stability of RSV Min L mentioned above [21], we found that the prominent missense mutations that Min L acquired during a temperature stress test paradoxically decreased rather than increased its replication in hamsters. Thus, mutations that appeared to be de-attenuating *in vitro* were not so *in vivo*. Furthermore, this paradoxically increased viral immunogenicity. These mutations also increased the genetic stability of Min L, thus yielding an improved live-attenuated RSV vaccine candidate that is currently being further investigated [43]. We used the same approach here on Min A and evaluated whether the missense P mutations might modify viral attenuation and/or improve Min A immunogenicity in rodents. We observed a broad effect of the P mutations on Min A replication in hamsters, with the mutation P[F28V] being of particular interest. Indeed, mutation P[F28V] paradoxically reduced Min A replication but increased its immunogenicity per PFU, thus generating an effect that was previously observed with a stabilized version of Min L.

The precise mechanism behind this increased immunogenicity is still unknown. A plausible explanation would be that CPD rRSVs, which typically contain increased frequencies of CpGs and UpAs dinucleotides, would provide greater stimulation–direct or indirect—of the activation and proliferation of B cells. During virus transcription and RNA replication, large amounts of double-stranded replicative intermediates are produced that could potentially be recognized by the innate immune response [44–46]. Both Min L and Min A viruses in which we re-introduced prominent missense mutations identified during the stress test exhibited increased transcription and RNA replication *in vitro*. Thus, it could be possible that these viruses generated increased amounts of viral RNA with increased frequencies of CpG and UpA dinucleotides that could stimulate the immune response more efficiently. This hypothesis is further supported by our previous study showing that RSVs that contained codon-pair optimized ORFs with decreased CpG and UpA content exhibited reduced immunogenicity in hamsters [47]. The effect of the codon-pair bias on the immune response will be evaluated in a future study.

We found that the introduction of the M ([K123M]) and L ([S2084P]) mutations in addition to the P[F28V] mutation further increased the genetic stability of Min A. Thus, Min A-P[F28V]-M[K123M]-L[S2084P] represents an improvement over Min A for several reasons: (i) it exhibited increased replication compared to Min A in Vero cells, which is important for vaccine manufacture, (ii) it did not accumulate additional mutations when passaged in stress tests at 39–40°C, and (iii), it was significantly more attenuated *in vivo* in the hamster model than Min A, and (iv) it was not significantly less immunogenic than Min A despite the increased attenuation.

Furthermore, Min A-P[F28V]-M[K123M]-L[S2084P] exhibits several advantages over the stabilized version of Min L that is currently being evaluated in a clinical trial. Even though Min L has been genetically stabilized in cell culture, its attenuation relies on the CPD of a single gene, L, while the attenuation of Min A relies on the CPD of six genes (NS1, NS2, N, P, M and SH) encoding for six proteins with very different functions. The involvement of multiple ORFs and their encoded proteins in the attenuation phenotype should be a more efficient barrier against reversion in humans.

CPD of the NS1 and NS2 ORFs and the resulting reduced expression of their encoded proteins was particularly noteworthy. Previous studies showed that deletion of NS1 and NS2 resulted in increased dendritic cell maturation and an increase in the expression of multiple cytokines and chemokines [48]. NS1 in particular was shown to have a suppressive effect on two cell populations, namely CD103+ CD8+ T cells and Th17 cells that are known to protect against viral respiratory infections [49]. Thus, reduced expression of NS1 and NS2 resulting from CPD might result in an increase in dendritic cell activation and maturation resulting in an increased quantity and quality of the immune response.

Since 2011, our laboratory has evaluated 12 live-attenuated RSV vaccine candidates in clinical trials [50–55]. This experience indicates that there is a range or “window” in which vaccine virus replication is sufficiently reduced to minimize reactogenicity without unacceptable loss of immunogenicity, and that this “window” is narrow. The difficulty in hitting this target is the reason why evaluation of multiple candidates has been necessary. Even relatively small differences in replication and immunogenicity between attenuated strains, have the potential to be important. Increasing the ratio of immunogenicity versus viral load, as observed in the present study, is particularly valuable. In summary, Min A-P[F28V]-M[K123M]-L[S2084P] is an improved vaccine candidate, appropriate for evaluation in a clinical study.

## Materials and methods

### Ethics statement

All animal studies were approved by the NIH Institutional Animal Care and Use Committee (IACUC).

### Cell lines

African green monkey kidney (Vero) cells were grown in OptiMEM (Gibco-Life Technologies) supplemented with 5% fetal bovine serum (FBS, Hyclone) and 1% L-glutamine (Gibco-Life Technologies) at 37°C with 5% CO_2_. Baby hamster kidney cells constitutively expressing the T7 polymerase (BSR T7/5) [56] were grown in GMEM media (Gibco-Life Technologies) supplemented with 10% FBS and 2% MEM amino acids (Gibco-Life Technologies). Every second cell passage, 2% of gentamicin (Quality Biological) was added to maintain selection for the T7-polymerase-expressing cells.

### Viruses

Min A was constructed in a previous work [20] and is described in the results. Its parent was RSV D46/6120, which is a derivative of wild-type (wt) RSV strain A2 (Genbank accession number KT992094) that contains a 112-nucleotide deletion in the downstream non-translated region of the SH gene and five silent nucleotide point mutations involving the last three codons and termination codon of the SH ORF [57]. These mutations stabilize the RSV cDNA during propagation in *E*. *coli* without affecting the replication of the recovered RSV *in vitro* and in mice [57]. Sequence numbering is based on recombinant wt RSV strain A2 (Genbank Accession number KT992094) containing the 112-nt deletion (nt 4499–4610 inclusive) noted above.

### Virus titration by immunoplaque assay

As previously described [20], 10-fold serial dilutions of virus were inoculated in duplicate in 24-well plates of Vero cells for two h at 32°C, and incubated at 32°C for seven to 10 days. The cells were fixed with 80% cold methanol, immunostained with a mixture of three RSV F-specific monoclonal antibodies (mAbs), and incubated with a polyclonal anti-mouse-IgG antibody linked to horseradish peroxidase for colorimetric visualization of plaques [20].

### Ion Torrent whole genome deep sequencing

Viral RNA was extracted from clarified cell-culture-medium supernatants collected at the end of the last passage (P18) using the QIAamp Viral RNA extraction kit (Qiagen) and reverse transcribed using Superscript II Reverse Transcriptase (RT, ThermoFisher). The cDNA was amplified by PCR using RSV-specific primers and a high-fidelity DNA polymerase (pfx DNA polymerase, Thermofisher) as described previously [21] and PCR amplicons were purified using the QIAquick PCR Purification kit (Qiagen). Then, Ion torrent deep sequencing was performed as previously described [21]. The only sequences that were not directly determined for each genome were the positions of the outer-most primers, namely nucleotides 1–23 and 15,174–15,223. DNA sequences were compared using VariantCaller 3.2 software (Ion Torrent). Parameters of the analysis pipeline were set at the Ion Torrent default somatic variant configuration. A nucleotide variant was called if the variant occurred >50 times with an average read depth of 1000 x and a P-value < 10^−7^ (Quality score >70) as previously described [21]. The raw read data were also manually verified using the IVG genome browser (The Broad Institute).

### Reverse genetics

Mutations were introduced into the Min A antigenomic cDNA using the QuikChange Lightning Site-Directed Mutagenesis Kit (Agilent Technologies). Mutant viruses were recovered by transfection into BSR-T7 cells followed by transfer to Vero cells (P1) as previously described [20,21]. A second passage was performed on Vero cells, the sequence of the resulting P2 virus stock was confirmed by Sanger and/or Ion Torrent deep sequencing of overlapping reverse transcribed PCR amplicons, and the P2 stocks were used in all experiments.

### Characterization of plaque size and F protein expression

Vero cell monolayers in six-well plates at 32°C were inoculated for two h with 250 PFU per well of virus, an overlay containing 0.8% methylcellulose was added to each well, and the cells were incubated for seven days and then fixed with 80% cold methanol. After overnight incubation in methanol, plates were incubated with a cocktail of three anti-RSV F mAbs [58] in Odyssey Blocking Buffer in PBS (Li-Cor), washed, and incubated with an R-phycoerythrin goat anti-mouse IgG(H+L) secondary antibody (Thermofisher). Plaques were visualized using the Celigo imager (Nexcelcom Bioscience). Images were analyzed using Celigo software to measure the plaque size (μm^2^) and the median fluorescence intensity (MFI) of F expression per plaque (i.e., the median value of all of the pixels per plaque). An average of 2140 (±1167) individual plaques were analyzed per virus in Fig 2B.

### Single-cycle infections

Single-cycle infections were performed in replicate monolayers of Vero cells in six-well plates as previously described [21]. Briefly, cells were infected at a MOI of three PFU/cell at 37°C with the indicated viruses. Two h after infection, the cell monolayers were washed twice with PBS to remove the inoculum. Every four h from four to 24 hpi, four wells per virus were harvested. As described in detail below, (i) one well was processed for cell-associated RNA for analysis by strand-specific RT-qPCR, (ii) cells from a second well were harvested for analysis by flow cytometry, (iii) a third well was processed for Western blot analysis, and (iv) a fourth well was used to quantify virus titer.

### Strand-specific RT-qPCR

Infected Vero cells from single-cycle infections (MOI of three PFU/well, 37°C, described above) were harvested and the cell-associated RNA was collected using the RNeasy Mini Kit (Qiagen). RNA was subjected to strand-specific RT-qPCR to quantify viral negative-sense (genome) and positive-sense (mRNA and antigenome) RNA, as described previously [20]. Viral RNA was extracted using the RNeasy Mini Kit (Qiagen), and five μg of DNAse-treated RNA was reverse transcribed using SuperScript III First-Strand Synthesis System (Thermofisher) with first-strand primer specific either to genome or to antigenomic/mRNA and linked to an oligonucleotide tag [21]. Then, each cDNA was amplified in triplicate with a primer containing the oligonucleotide tag, a gene-specific reverse primer, and a probe. Strand-specificity was provided because only cDNAs containing the tagged RT primer sequence would be amplified. QPCR results were analyzed using the comparative threshold cycle (ΔCt) method, normalized to 18S rRNA internal control that had been subjected to RT-qPCR using random first-strand primers and a standard 18S rRNA Taqman assay (Thermofisher). Data were expressed as log_2_ fold increase over the Min A four-h time point except for the quantification of wt NS1, NS2, N, P, M, and SH genes in wt RSV-infected cells that were expressed as fold-increase over wt four-h time point.

### Flow cytometry

Infected Vero cells from single-cycle infections (MOI of three PFU/cell, 37°C, described above) were harvested using TrypLE Select (Gibco) and stained with Live/Dead Fixable Near-IR Dead Cell dye (Thermofisher), followed by fixation and permeabilization using BD Cytofix/Cytoperm (BD Biosciences). Fixed and permeabilized cells in Perm/Wash buffer (BD Biosciences) were stained with a mixture of anti-RSV antibodies for the analysis of intracellular RSV protein expression: a fluorescein isothiocyanate (FITC)-labeled anti-RSV P mAb (Abcam), an allophycocyanin (APC)-labeled anti-RSV N mAb (Imgenex), and a Biotin-labeled anti-RSV F mAb (Millipore). Staining was performed for 30 min at room temperature in the dark. After incubation with the primary antibodies, cells were extensively washed with Perm/Wash Buffer (BD Biosciences) and then incubated with a pre-titrated concentration of streptavidin-PE secondary antibody in the dark for 20 min at room temperature. Live single cells were acquired using a BD flow cytometer Symphony (BD Biosciences). Data were analyzed using FlowJo 10.7. First, quality control of each acquired sample was performed using the FlowAI plugin that evaluates the flow rate, signal acquisition and dynamic range and removes cells with identified anomalies [59]. Then, compensation was performed automatically using single-color-labeled cells or beads for each antibody. Live/dead staining, forward scatter height, and forward scatter area were used to identify single live cells. Finally, the cell number was normalized to 19,000 across all samples using the DownSample plugin (FlowJo 10.7) and the expression of the virus proteins N, P, and F was analyzed on single live cells.

### Western blot analysis

Infected Vero cells from single-cycle infections (MOI of three PFU/cell, 37°C, described above) were harvested in NuPage LDS sample buffer (Thermofisher) followed by homogenization using a QIAshredder spin column (Qiagen). Cell lysates were denatured at 90°C for 10 min in 1X NuPAGE LDS Sample Buffer (Invitrogen) and 1X NuPAGE Sample Reducing Agent (Invitrogen) and subjected to electrophoresis in parallel with Odyssey Protein Molecular Weight Markers (Li-Cor) on NuPAGE 4–12% Bis-Tris Protein Gels (Thermofisher) with NuPAGE MES SDS Running Buffer (Life Technologies). Proteins were transferred to PVDF membranes using the iBlot 2 Gel Transfer Device (ThermoFisher). Membranes were blocked using Odyssey Blocking Buffer for one h followed by overnight incubation with primary antibodies in Odyssey Blocking Buffer in PBS with 0.1% Tween 20 (Sigma-Aldrich). The primary antibodies were mouse mAbs against RSV N, P, M2-1 and G proteins (1:1,000, Abcam) and a rabbit polyclonal antibody preparation against GAPDH (1:200, Santa Cruz) as a loading control. The secondary antibodies used were goat anti-rabbit IgG IRDye 680, and goat anti-mouse IgG IRDye 800 (1,15000, Li-Cor). Membranes were scanned using Odyssey software, version 3.0 (Li-Cor). Fluorescence signals of the RSV protein bands were corrected to subtract the background signal from the membrane just outside of each band by the Image Studio Lite software (Licor). Values indicate the fluorescence intensity (FI) of each protein band.

### Animal experiments

Replication, immunogenicity, and protective efficacy of the CPD viruses was evaluated in six-week old Golden Syrian hamsters in two separate experiments.

On day 0, groups of 14 and 18 hamsters, in experiment #1 and #2, respectively, were inoculated intranasally under isoflurane anesthesia with 10^6^ PFU of wt RSV, Min A, or the indicated Min A-derived viruses. In each experiment, three additional hamsters were left uninfected as control. On day three, which corresponds to the peak of replication of wt RSV in hamsters, half of the hamsters from each inoculated group were euthanized by carbon dioxide inhalation. Nasal turbinates (NT) and lungs were harvested and homogenized separately in Leibovitz (L-15) medium containing 2% L-glutamine, 1% Amphotericin B, 0.1% Gentamicin, and 0.06 mg/mL clindamycin phosphate. Virus titers were determined in duplicate by immunoplaque assay on Vero cells incubated in 32°C. The limit of virus detection was 50 PFU/g in both the NT and lungs.

Immunogenicity of CPD viruses was also tested. Serum was collected from the blood of seven and nine hamsters per group the day prior to immunization in experiment #1 and #2, respectively, and at day 24 and 25 post-immunization in experiment #1 and #2, respectively, to measure the RSV antibody response. The PRNT_60_ were determined as described previously [20].

On day 27 and 28 post-immunization in experiment #1 and #2, respectively, the remaining hamsters were challenged with 10^6^ PFU of wt RSV via intranasal administration. Three days after challenge, hamsters were euthanized by carbon dioxide inhalation. NT and lung tissue were harvested and wt RSV virus titers were determined in duplicate by plaque assay on Vero cells incubated at 32°C as described above.

### Molecular dynamics (MD) simulations of P40 and the N^0^-P complex

In previous work by others [37], low-resolution models of a truncated soluble form of the RSV N protein and a peptide representing amino acids 1–40 of the RSV P protein (P40) were created from small-angle x-ray scattering experiments (SAXS) and analytical ultracentrifugation (AUC). We used initial conformations of the RSV N-P40 complex from the constructs reported in [37]. These published models were based on versions of N and P40 that had been modified to facilitate computation: specifically, the N protein (i) had a deletion of residues 1–30, (ii) had aa 250–255 (which forms a crevice between the NTD [aa 31–249] and CTD [aa 256–359] of N^0^) replaced with six Gly residues, and (iii) had aa 360–373 in the C-terminal arm [aa 360–391] replaced with 15 Gly residues; and in P40, the C-terminal 14 residues had been replaced with 14 Gly residues. In the present study, we used the published structures [37] together with the complete authentic sequences of RSV N and P40 of strain A2 (which are identical in sequence to N and P40 of the closely-related Long strain used in the published work) to create complete atomic-resolution models of the N^0^-P40 complex in aqueous solution.

Short simulations were first performed for the N^0^ and P40 monomers in isolation to allow the structures of the newly created atomic-resolution models to relax to the molecular forcefield and physiological conditions. Snapshots of each relaxed molecule at the end of the simulations were then used to create the initial models of the N^0^-P40 complexes (this was done by positioning the α-helix of P40 relative to CTD as it appears in the low-resolution models; this was done in practice by superimposing the C_α_ atoms). These structures were used as starting point in all the simulations. All MD simulations were performed with the CHARMM program [60], using the TIP3P water model and the CHARMM36 protein forcefield [61], with particle-mesh Ewald summations and cubic periodic boundary conditions, at 35°C, one atm, in 120 mM potassium chloride, and protonation states consistent with neutral pH. All the *in silico* settings were initially energy-minimized, heated, and equilibrated following standard protocol. Production times varied between 20 ns and 100 ns, depending on the system size and purpose of the analysis. Patterns of hydrogen bonds (H-bonds), salt bridges, and hydrophobic contacts were calculated intra- (for P40 in the N^0^-P complex) and inter-molecularly (for the N^0^-P complex) using standard donor-acceptor and carbon-carbon distance criteria. Interactions observed with high frequencies within each independent simulation were considered statistically significant and determinant of the system behavior.

### Statistical analysis

Distributions of the virus plaque area and F MFI in Fig 2 were compared for statistical significance using the ANOVA test. Sets of data were only considered statistically different at p≤0.05. Data sets obtained from the quantification of the protein expression by western blot or virus titers by plaque assay (Fig 4C and 4E) and from the hamster experiments (Figs 5 and S4) were assessed for significance using parametric one-way repeated measures ANOVA with the Tukey post hoc tests for normally distributed data sets or the non-parametric Kruskall Wallis test with Dunns post hoc test for non-normal data sets. A log_10_ transformation was applied to data sets when necessary to obtain equal standard deviations among groups, a necessary requirement of both tests. Statistics were performed on the Prism 8 version GraphPad Software). Data were only considered significant at p≤0.05.

## Supporting information

S1 Table Mutations detected at a frequency of ≥5% in each of the nine Min A lineages at the end of the temperature stress test as well as in each of the two controls. (DOCX)

S1 Fig The P mutations restored RNA synthesis by Min A during a single-cycle infection (additional data from the experiment in Fig 3). As an additional part of the experiment shown in Fig 3, additional replicate cultures of Vero cells infected with the indicated viruses (MOI three PFU/cell, 37°C) were harvested at four-h intervals from four to 24 hpi and processed for intracellular RNA. The RNA was analyzed by RT-qPCR specific for positive-sense (mRNA and antigenome) NS1, NS2, M and SH RNAs. Results were normalized to internal 18S rRNA. Data are expressed as fold-increase over the result for wt RSV at the four-h time point for wt genes (solid lines) and as fold-increase over the result for Min A at the four-h time point for CPD genes (dashed lines). All of the ORFs shown here for Min A and its derivatives were CPD; thus, as described in the legend to Fig 3 and the Materials and Methods, the extensive sequence differences between the wt and CPD ORFs necessitated the use of two different sets of primers/probes for wt RSV versus CPD ORFs. Because of this, direct comparison between the wt and CPD ORFs cannot be made for the data shown here. However, Fig 3 includes data for other ORFs that were wt in both wt RSV and the Min A viruses, and used the same primers and probes, and thus those data can be directly compared.
(TIF)

S2 Fig Temperature stress test of Min A-P[K27N] and Min A-P[F28V]. The stability of Min A-P[K27N] and Min A-P[F28V] was evaluated in a temperature stress test. Five replicate cultures of Vero cells in 25 cm^2^ flasks were inoculated with an MOI of 0.1 PFU/cell. Three replicate cultures (left panel) were incubated at 39°C for four passages and 40°C for an additional four passages, representing two months of culture. The remaining two replicate cultures (right panel) were passaged in parallel for eight passages at the permissive temperature of 32°C. Flasks were harvested when extensive syncytia were observed or when the cells started to detach. Clarified fluids from the previous passage were used to infect the following passage of fresh cells in a 1:5 dilution. In addition, aliquots of clarified virus from each lineage were snap frozen for virus titration by plaque assay at 32°C. Whole genome Sanger sequencing was performed at the end of the experiment (P8).
(TIF)

S3 Fig Generation of additional Min A derivatives and evaluation of their replication *in vitro*. (A) Gene map of Min A and Min A derivatives. CPD genes are shown in black, while wt ORFs are shown in grey. Two prominent mutations (a1138g and L[V151A]) identified in lineage #4 that also contained the prominent P[K27N] mutation (see Table 1) were re-introduced by site-directed mutagenesis into Min A-P[K27N] cDNA to generate the Min A-P[K27N]+2 virus. Five prominent mutations (t612c, c2334t, a3195g, M[K123M] and L[S2084P]) identified in lineage #5 that also contained the prominent P[F28V] mutation (see Table 1) were re-introduced in two different combinations by site-directed mutagenesis into Min A-P[F28V] cDNA to generate the Min A-P[F28V]+2 and Min A-P[F28V]+5 viruses. These three viruses were rescued by reverse genetics and their respective sequences were confirmed by Sanger sequencing. (B, C) The replication of the Min A-P[K27N]+2, Min A-P[F28V]+2, and Min A-P[F28V]+5 viruses was evaluated in a multicycle replication experiment in Vero cells infected using an MOI of 0.01 PFU/cell and incubated at 32°C (left) or 37°C (right). Wt RSV, Min A, P16 of lineage #4 and #6 were used for comparison. Duplicate wells for wt RSV, Min A, and the Min A-derivatives were harvested daily. Virus titers were determined by immunoplaque assay and are shown as means with standard deviation of two replicate titrations of two replicates at each timepoint. Due to limited samples, titers for P16 viruses correspond to the mean of two replicate titrations with the standard deviation of one sample at each timepoint. Day 0 titers correspond to the back titration of the inocula.
(TIF)

S4 Fig Replication, immunogenicity, and protective efficacy of Min A-P[K27N]+2, Min A-P[F28V]+2, and Min A-P[F28V]+5 viruses *in vivo*. Groups of 18 six-week-old golden Syrian hamsters were inoculated intranasally with 10^6^ PFU of the indicated virus per animal. Three hamsters were left uninfected as control. (A) **Replication**. Nasal turbinates (NT) and lungs were harvested at day three pi from nine hamsters per group, evaluated by immunoplaque assay, and expressed as PFU/g of tissue. The limit of detection, 50 PFU/g, is indicated by a dotted line. (B) **Immunogenicity**. Titers of serum RSV-neutralizing antibodies at day 25 pi were determined from nine hamsters per group. The PRNT_60_ in log_2_ are shown. (C) **Protective efficacy.** At day 28 pi, nine hamsters per group and the three control hamsters were inoculated intranasally with 10^6^ PFU of wt RSV. Three days after challenge, NT and lungs were harvested and titers of challenge wt RSV were determined by immunoplaque assay. In each graph, each hamster is represented by a colored circle and the median value and standard deviation are shown with bars. The number of hamsters with replicating virus is indicated. In panel A, statistical differences are indicated at the top of each graph in comparison to wt RSV, while differences between Min A and Min A derivatives are indicated in brackets. In panel B, all statistical differences identified are against wt RSV (*p ≤ 0.05; **p ≤ 0.01; ***p ≤ 0.001; ****p ≤ 0.0001; ns = non-significant).
(TIF)
