# Supplementary material for: Reversion mutations in phosphoprotein P of a codon-pair-deoptimized human respiratory syncytial virus confer increased transcription, immunogenicity, and genetic stability without loss of attenuation
Source: PLoS Pathog. 2021 Dec 29;17(12):e1010191. doi: 10.1371/journal.ppat.1010191 (PMC8751989; doi:10.1371/journal.ppat.1010191)
Supplement: S1 Table — (DOCX) [file ppat.1010191.s001.docx]

**S1 Table.** Mutations detected at a frequency of ≥5% in each of the nine Min A lineages at the end of the temperature stress test as well as in each of the two controls.

|  |  |  | **Lineage no. and percentage of reads with the indicated mutation*** | | | | | | | | | | |
| --- | --- | --- | --- | --- | --- | --- | --- | --- | --- | --- | --- | --- | --- |
| **Gene** | **nt mutation** | **aa mutation** | **1** | **2** | **3** | **4** | **5** | **6** | **7** | **8** | **9** | **Ct1** | **Ct2** |
| NS1 | t440g^†‡^ | S114R |  |  |  |  |  |  | 6 |  |  |  |  |
| NS2 5’UTR | t612c | / |  |  |  |  | 91 |  |  |  |  |  |  |
| NS2 | a723g | R32R (silent) |  |  |  | 9 |  |  |  |  |  |  |  |
| N 5’UTR | a1138g | / |  |  |  | 59 |  |  |  |  |  |  |  |
| N | c1301a^†^ | T54N |  |  |  |  |  |  |  | 49 |  |  |  |
| N | t1380c^†‡^ | I80I (silent) |  |  |  |  |  | 7 |  |  |  |  |  |
| N | t1410c^†‡^ | V90V (silent) |  |  |  |  |  | 6 |  |  |  |  |  |
| N | a1459c^†^ | K107Q |  | 57 |  |  |  |  |  |  |  |  |  |
| N | a1460g^†^ | K107R | 7 |  |  |  |  |  |  |  |  |  |  |
| N | a1547g^†^ | K136R |  |  | 42 |  |  |  |  |  |  |  |  |
| N | a1749g^†‡§^ | K203K (silent) |  |  |  | 94 |  |  |  |  |  |  |  |
| N | c1929t^†‡^ | L263L (silent) |  |  |  |  |  | 18 |  |  |  |  |  |
| N | t2151c^†‡§^ | Y337Y (silent) |  |  |  |  |  |  |  | 62 |  |  |  |
| P gene start | c2334t | / |  |  |  |  | 68 |  |  |  |  |  |  |
| P | t2364c | P6P (silent) |  |  |  |  |  |  | 64 |  | 61 |  |  |
| P | a2376t | G10G (silent) |  |  |  | 77 |  |  |  |  |  |  |  |
| P | t2388c^†‡§^ | N14N (silent) |  |  |  |  |  |  |  |  | 45 |  |  |
| P | a2402t | K19I |  |  |  |  |  | 44 |  |  |  |  |  |
| P | t2405a | F20Y |  |  |  |  |  |  |  |  | 42 |  |  |
| P | a2419g | K25E |  |  |  |  |  |  |  |  |  |  | 36 |
| P | a2420c | K25T | 98 | 94 |  |  |  |  |  |  |  |  |  |
| P | g2421t | K25N |  |  |  |  |  |  |  |  |  | 58 | 49 |
| P | t2424c^†‡§^ | G26G (silent) |  |  |  |  |  |  |  | 10 |  |  |  |
| P | a2425g^†^ | K27E |  |  |  |  |  |  |  | 11 |  | 33 |  |
| P | g2427t^†‡^ | K27N |  |  |  | 97 |  |  |  | 62 |  |  |  |
| P | t2428g^†^ | F28V |  |  |  |  | 98 |  |  | 13 | 53 |  |  |
| P | t2428a^†^ | F28I |  |  |  |  |  | 24 |  |  |  |  |  |
| P | t2428c^†^, t2429c^†^ | F28P |  |  |  |  |  |  | 58 |  |  |  |  |
| P | a2441c | K32T |  |  | 93 |  |  |  |  |  |  |  |  |
| P | c2446t^†^ | P34S |  |  | 92 |  |  |  |  |  |  |  |  |
| P | t2577c^†‡§^ | P77P (silent) |  | 27 |  |  |  |  | 73 |  |  |  |  |
| P | g2665a | E107K |  |  |  |  |  |  |  | 13 |  |  |  |
| P | g3032a^†^ | G229E |  |  | 91 |  |  |  |  |  |  |  |  |
| P | a3046c^†^ | N234H | 78 |  |  |  |  |  |  |  |  |  |  |
| P | a3050g | D235G |  |  |  | 8 |  |  |  |  |  |  |  |
| P 3’UTR | a3195g | / |  |  |  |  | 89 |  |  |  |  |  |  |
| M | a3629t^†^ | K123M |  |  |  |  | 62 |  |  |  |  |  |  |
| M | g3770a | R170K |  |  |  |  |  |  |  |  | 15 |  |  |
| M-SH intergenic | t4211a | / |  |  |  |  | 9 |  |  |  |  |  |  |
| SH | t4351c | P16P (silent) |  |  |  |  |  |  |  |  |  | 12 |  |
| SH | t4352c | Y17H |  |  |  |  |  |  |  |  |  | 12 |  |
| SH | t4411c^†‡§^ | I36I (silent) |  |  |  |  |  |  |  |  |  | 12 |  |
| SH | t4426c^†‡^ | L41L (silent) |  |  |  |  |  |  |  |  |  | 12 |  |
| SH | t4449c | V49A |  |  |  |  |  |  |  |  |  | 11 |  |
| SH gene end | t4619c | / |  |  |  |  |  |  |  |  |  | 12 |  |
| SH gene end | t4620c | / |  |  |  |  |  |  |  |  |  | 13 |  |
| SH-G intergenic | a4662g | / |  |  |  |  |  |  |  | 53 |  |  |  |
| G | c5141t | R151R (silent) |  |  |  |  |  |  |  |  |  | 15 |  |
| G | a5167g | N160S |  |  |  |  |  |  |  |  |  |  | 17 |
| G | a5169g | N161D |  |  |  |  |  |  |  |  |  |  | 18 |
| G | a5170g | N161S |  |  |  |  |  |  |  |  |  |  | 30 |
| G | a5194g | N169S |  |  |  |  |  |  |  |  |  |  | 21 |
| G | a5424g | T246A |  |  |  |  |  |  |  |  |  |  | 23 |
| G | a5429g | L247L (silent) |  |  |  |  |  |  |  |  |  |  | 25 |
| G | a5477g | E263E (silent) |  |  |  |  |  |  |  |  |  |  | 13 |
| G | a5478g | T264A |  |  |  |  |  |  |  |  |  |  | 14 |
| F | a6996t | K445N |  |  |  | 9 |  |  |  |  |  |  |  |
| F | a7163g | Q501R |  |  |  |  |  |  |  |  | 14 |  |  |
| F | a7194g | E511E (silent) |  |  |  |  |  |  |  | 6 |  |  |  |
| M2-1 | a7616g | R4G |  |  |  |  | 38 |  |  |  |  |  |  |
| M2-1 | a7647g | H14R |  |  |  |  |  |  |  | 8 |  |  |  |
| M2-1 | t7674c | F23S |  |  |  |  | 8 |  |  |  |  |  |  |
| M2-1 | a7754g | M50V |  |  |  |  |  |  |  |  | 78 |  |  |
| M2-1 | a8091g | K162R |  |  |  |  |  | 54 |  |  |  |  |  |
| M2-1 | a8134t | P176P (silent) |  |  |  | 7 |  |  |  |  |  |  |  |
| M2-2 | c8227t | T23I |  |  |  |  | 7 |  |  |  |  |  | 20 |
| M2-2 | t8343c | S62P |  |  |  |  |  |  |  |  |  |  |  |
| M2-2 | t8356g | I66S |  |  |  | 5 |  |  |  |  |  |  |  |
| L | t8950c, t8951c | V151A |  |  |  | 59 |  |  |  |  |  |  |  |
| L | t9453c | C319R |  | 9 |  |  |  | 56 |  | 11 |  |  |  |
| L | a10434t | M646L |  | 6 |  |  |  |  |  |  |  |  |  |
| L | t10622c | F708F (silent) |  |  |  |  |  |  |  | 45 |  |  |  |
| L | a10782g | N762D | 77 |  |  |  |  |  |  |  |  |  |  |
| L | a11361t | I955L |  |  |  |  |  |  |  | 49 |  |  |  |
| L | g11574a | D1026N | 99 | 97 |  |  |  |  |  |  |  |  |  |
| L | a12435t | S1313C |  |  |  |  |  |  | 9 |  |  |  |  |
| L | g13194a | V1566I |  |  |  |  |  |  | 69 |  |  |  |  |
| L | a13208g | I1570M |  |  |  |  |  |  |  | 58 |  |  |  |
| L | g13679a | K1727K (silent) |  |  |  |  |  |  |  | 11 |  |  |  |
| L | t13739c | S1747S (silent) |  |  |  |  |  | 18 |  |  |  |  |  |
| L | t13753c | I1752T |  |  |  |  |  | 19 |  |  |  |  |  |
| L | t13754c | I1752I (silent) |  |  |  |  |  | 19 |  |  |  |  |  |
| L | t13797c | L1767L (silent) |  |  |  |  |  | 12 |  |  |  |  |  |
| L | a13898g | I1800M |  |  |  |  |  |  | 69 |  |  |  |  |
| L | t14105c | P1869P (silent) |  |  |  |  |  |  | 32 |  |  |  |  |
| L | t14276c | F1926F (silent) |  |  |  |  |  |  |  |  |  | 12 |  |
| L | t14669c | F2057F (silent) |  |  |  |  |  | 68 |  |  |  |  |  |
| L | t14748c | S2084P |  |  | 62 |  | 93 |  |  |  |  |  |  |
| 5’ end trailer | t15163c | / |  |  |  | 7 |  |  |  |  |  |  |  |

“/” indicates that the amino acid mutation is not applicable for this particular mutation as the given mutation is localized in a non-translated region.

*Percentage of reads with the indicated mutation; only mutations present in ≥5% of the reads are shown.

Mutations detected in ≥45% of the reads are highlighted in yellow.

Mutations identified between aa 25 and 34 inclusive of P are highlighted by a grey shading.

Note that mutations t2428c and t2429c, and mutations t8950c and t8951c were each colocalized on the same viral genomes.

Nucleotide numbering is based on RSV sequence KT992094.

^†^Mutations involving a codon that had been changed as part of CPD of NS1, NS2, N, P, M, or SH.

^‡^Mutations involving a nucleotide that had been changed as part of CPD of NS1, NS2, N, P, M, or SH.

^§^Mutation involving a nucleotide that had been changed as part of CPD of NS1, NS2, N, P, M, or SH and that restored WT sequence.
