# Supplementary figures and images for: Reversion mutations in phosphoprotein P of a codon-pair-deoptimized human respiratory syncytial virus confer increased transcription, immunogenicity, and genetic stability without loss of attenuation
Source: PLoS Pathog. 2021 Dec 29;17(12):e1010191. doi: 10.1371/journal.ppat.1010191 (PMC8751989; doi:10.1371/journal.ppat.1010191)

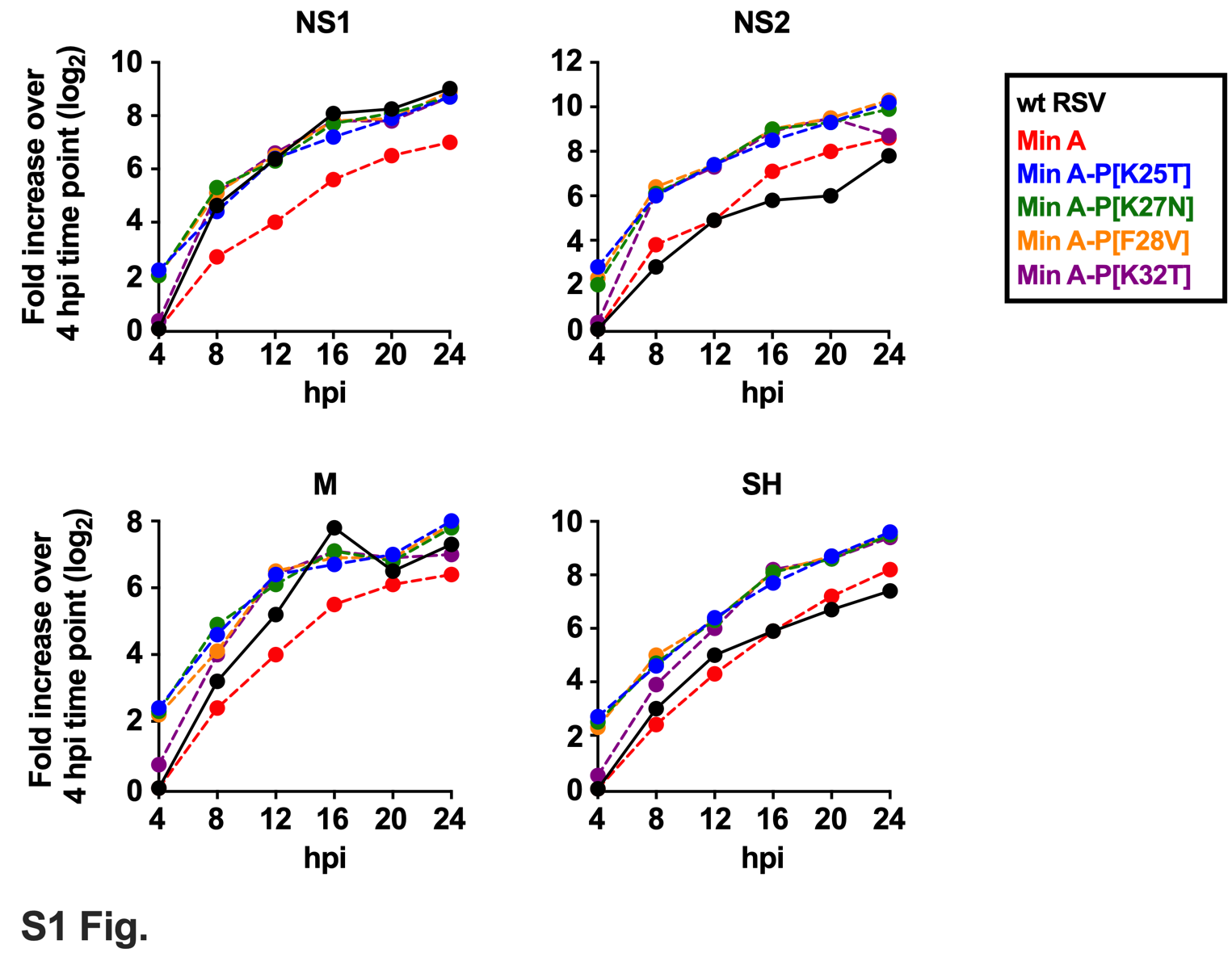

Supplement: S1 Fig — As an additional part of the experiment shown in Fig 3, additional replicate cultures of Vero cells infected with the indicated viruses (MOI three PFU/cell, 37°C) were harvested at four-h intervals from four to 24 hpi and processed for intracellular RNA. The RNA was analyzed by RT-qPCR specific for positive-sense (mRNA and antigenome) NS1, NS2, M and SH RNAs. Results were normalized to internal 18S rRNA. Data are expressed as fold-increase over the result for wt RSV at the four-h time point for wt genes (solid lines) and as fold-increase over the result for Min A at the four-h time point for CPD genes (dashed lines). All of the ORFs shown here for Min A and its derivatives were CPD; thus, as described in the legend to Fig 3 and the Materials and Methods, the extensive sequence differences between the wt and CPD ORFs necessitated the use of two different sets of primers/probes for wt RSV versus CPD ORFs. Because of this, direct comparison between the wt and CPD ORFs cannot be made for the data shown here. However, Fig 3 includes data for other ORFs that were wt in both wt RSV and the Min A viruses, and used the same primers and probes, and thus those data can be directly compared. (TIF) [file ppat.1010191.s002.tif]

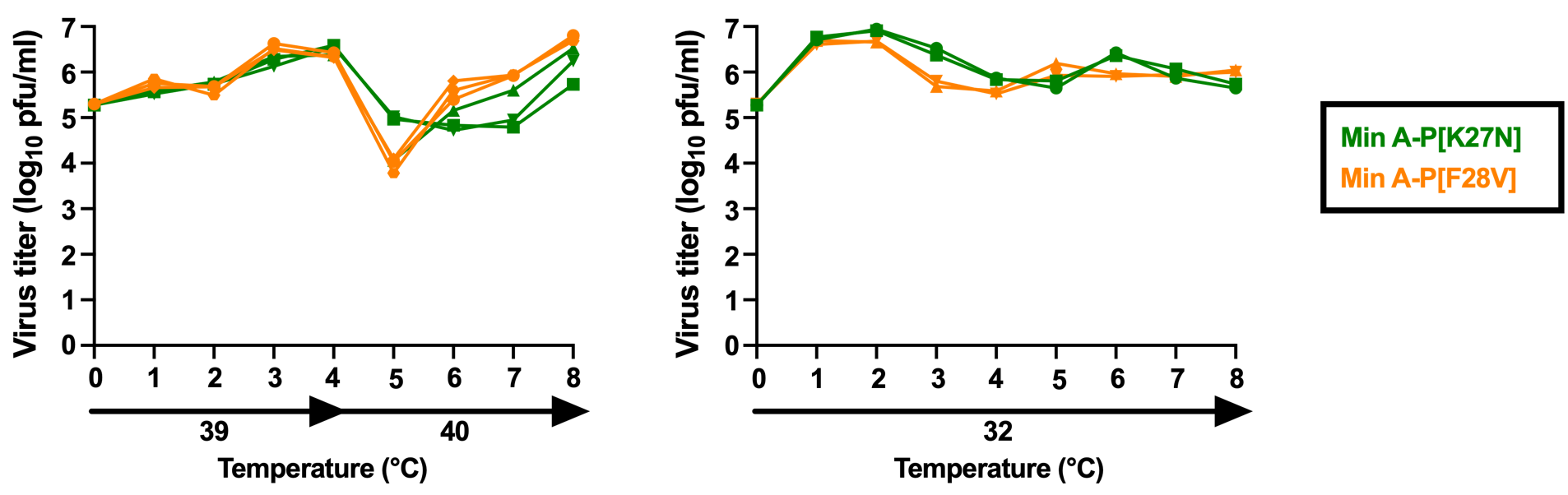

Supplement: S2 Fig — The stability of Min A-P[K27N] and Min A-P[F28V] was evaluated in a temperature stress test. Five replicate cultures of Vero cells in 25 cm2 flasks were inoculated with an MOI of 0.1 PFU/cell. Three replicate cultures (left panel) were incubated at 39°C for four passages and 40°C for an additional four passages, representing two months of culture. The remaining two replicate cultures (right panel) were passaged in parallel for eight passages at the permissive temperature of 32°C. Flasks were harvested when extensive syncytia were observed or when the cells started to detach. Clarified fluids from the previous passage were used to infect the following passage of fresh cells in a 1:5 dilution. In addition, aliquots of clarified virus from each lineage were snap frozen for virus titration by plaque assay at 32°C. Whole genome Sanger sequencing was performed at the end of the experiment (P8). (TIF) [file ppat.1010191.s003.tif]

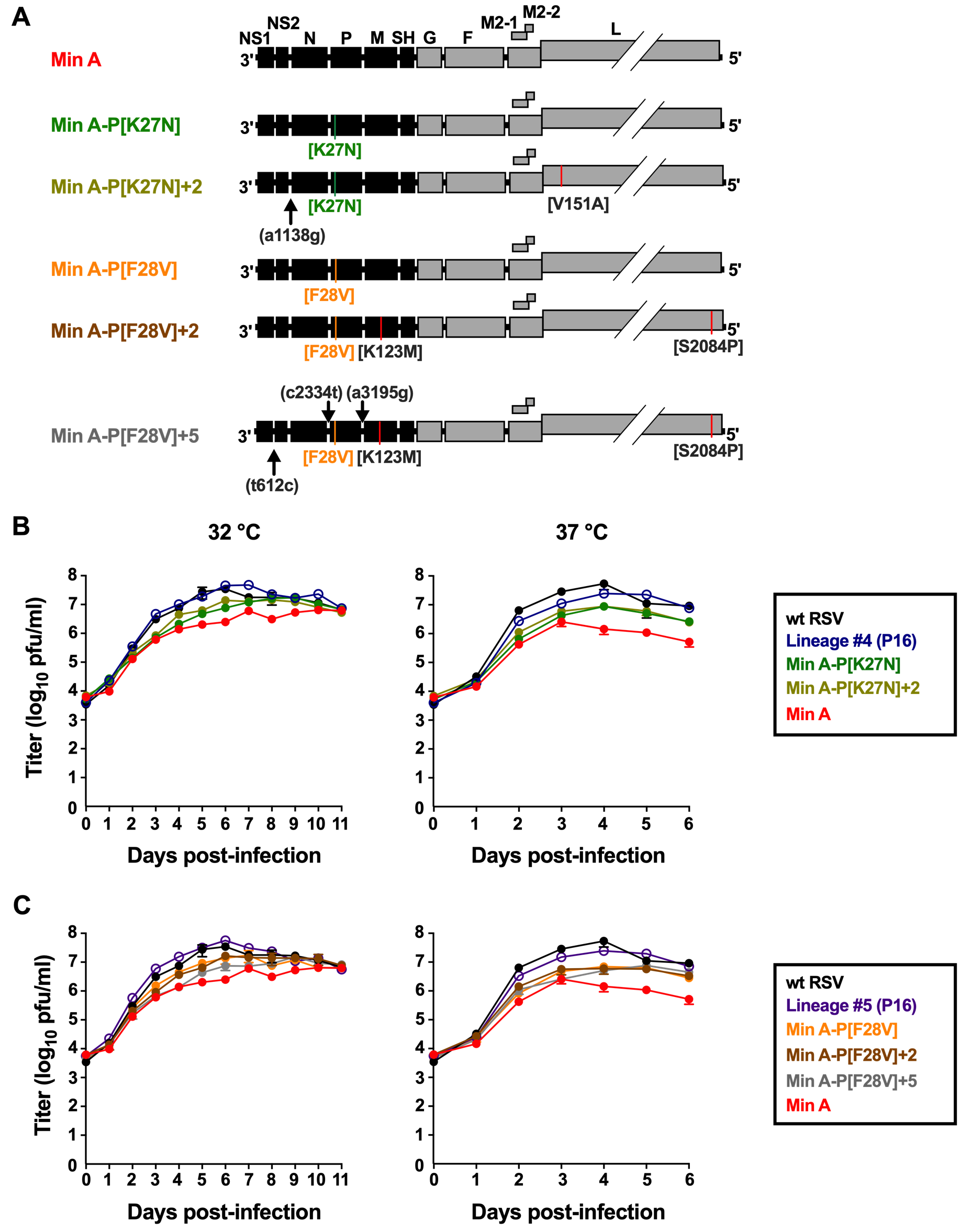

Supplement: S3 Fig — (A) Gene map of Min A and Min A derivatives. CPD genes are shown in black, while wt ORFs are shown in grey. Two prominent mutations (a1138g and L[V151A]) identified in lineage #4 that also contained the prominent P[K27N] mutation (see Table 1) were re-introduced by site-directed mutagenesis into Min A-P[K27N] cDNA to generate the Min A-P[K27N]+2 virus. Five prominent mutations (t612c, c2334t, a3195g, M[K123M] and L[S2084P]) identified in lineage #5 that also contained the prominent P[F28V] mutation (see Table 1) were re-introduced in two different combinations by site-directed mutagenesis into Min A-P[F28V] cDNA to generate the Min A-P[F28V]+2 and Min A-P[F28V]+5 viruses. These three viruses were rescued by reverse genetics and their respective sequences were confirmed by Sanger sequencing. (B, C) The replication of the Min A-P[K27N]+2, Min A-P[F28V]+2, and Min A-P[F28V]+5 viruses was evaluated in a multicycle replication experiment in Vero cells infected using an MOI of 0.01 PFU/cell and incubated at 32°C (left) or 37°C (right). Wt RSV, Min A, P16 of lineage #4 and #6 were used for comparison. Duplicate wells for wt RSV, Min A, and the Min A-derivatives were harvested daily. Virus titers were determined by immunoplaque assay and are shown as means with standard deviation of two replicate titrations of two replicates at each timepoint. Due to limited samples, titers for P16 viruses correspond to the mean of two replicate titrations with the standard deviation of one sample at each timepoint. Day 0 titers correspond to the back titration of the inocula. (TIF) [file ppat.1010191.s004.tif]

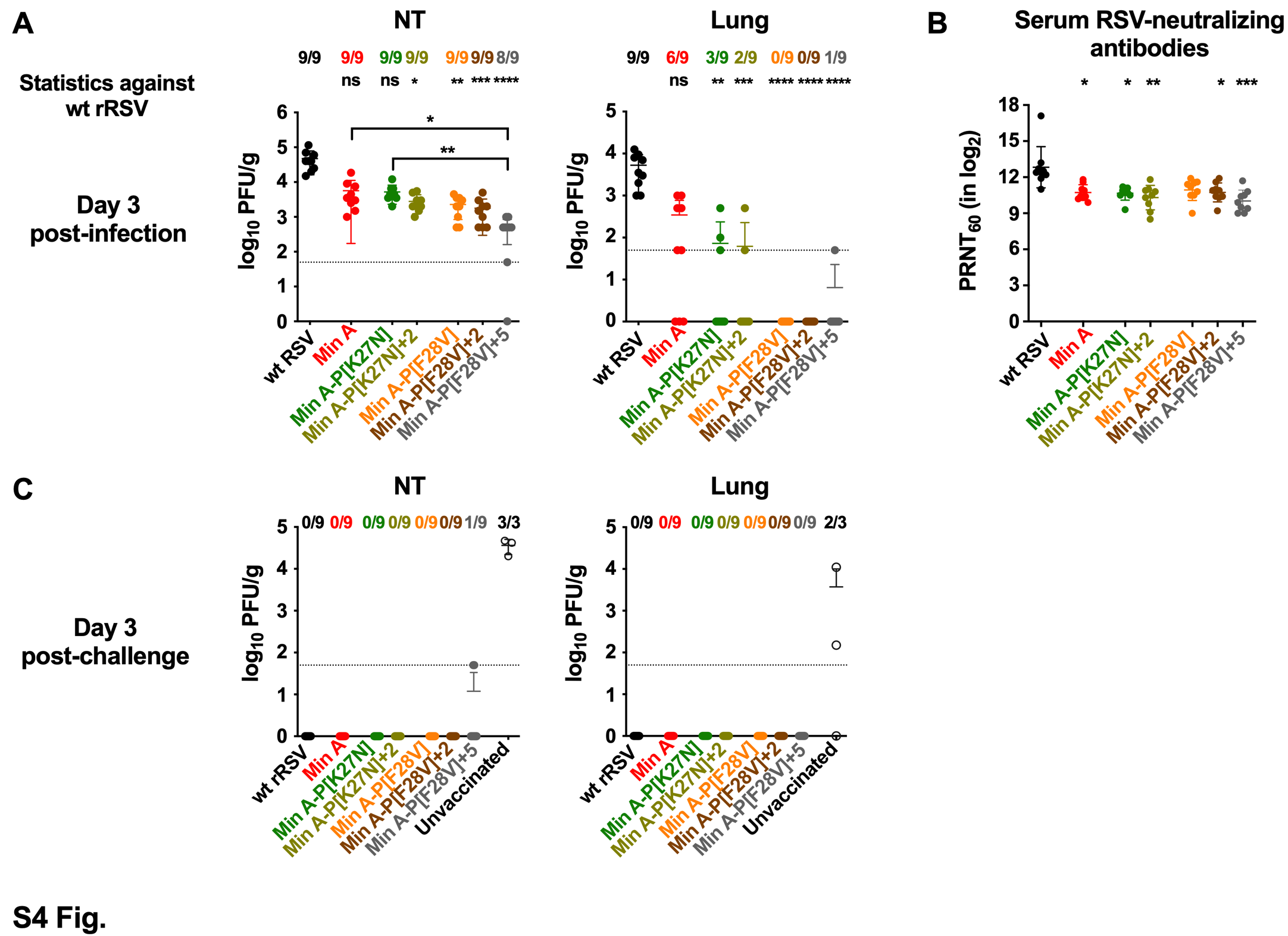

Supplement: S4 Fig — Groups of 18 six-week-old golden Syrian hamsters were inoculated intranasally with 106 PFU of the indicated virus per animal. Three hamsters were left uninfected as control. (A) Replication. Nasal turbinates (NT) and lungs were harvested at day three pi from nine hamsters per group, evaluated by immunoplaque assay, and expressed as PFU/g of tissue. The limit of detection, 50 PFU/g, is indicated by a dotted line. (B) Immunogenicity. Titers of serum RSV-neutralizing antibodies at day 25 pi were determined from nine hamsters per group. The PRNT60 in log2 are shown. (C) Protective efficacy. At day 28 pi, nine hamsters per group and the three control hamsters were inoculated intranasally with 106 PFU of wt RSV. Three days after challenge, NT and lungs were harvested and titers of challenge wt RSV were determined by immunoplaque assay. In each graph, each hamster is represented by a colored circle and the median value and standard deviation are shown with bars. The number of hamsters with replicating virus is indicated. In panel A, statistical differences are indicated at the top of each graph in comparison to wt RSV, while differences between Min A and Min A derivatives are indicated in brackets. In panel B, all statistical differences identified are against wt RSV (*p ≤ 0.05; **p ≤ 0.01; ***p ≤ 0.001; ****p ≤ 0.0001; ns = non-significant). (TIF) [file ppat.1010191.s005.tif]
